# Neuromuscular Characteristics Associated with Knee Instability in Osteoarthritis and After Total Knee Replacement: A Systematic Review and Meta-Analysis

**DOI:** 10.3390/clinpract16040074

**Published:** 2026-04-14

**Authors:** Ariane P. Lallès, Luisa Cedin, Markus A. Wimmer

**Affiliations:** 1 Department of Orthopedic Surgery, Rush University Medical Center, 1611 W Harrison Street, Suite 201, Chicago, IL 60612, USA; 2 Arts et Metiers Institute of Technology, EPF Engineering School, Université Sorbonne Paris Nord, IBHGC-Institut de Biomécanique Humaine Georges Charpark, F-75013 Paris, France; ariane.lalles@ensam.eu

**Keywords:** knee instability, knee laxity, muscle co-contraction, muscle strength, total knee arthroplasty, knee osteoarthritis

## Abstract

**Background/Objectives**: Knee joint instability is frequently reported in individuals with knee osteoarthritis (OA) and may persist after total knee replacement (TKR), where it represents a leading cause of revision. However, neuromuscular factors associated with knee instability remain poorly understood. This systematic review and meta-analysis aimed to compare neuromuscular characteristics between individuals with stable and unstable knees in OA and TKR populations. **Methods**: PubMed, CENTRAL, Scopus, and EMBASE were searched from inception to 10 January 2025. Studies comparing neuromuscular outcomes between stable and unstable knees were included. Neuromuscular parameters included: muscle strength, muscle power, muscle activation pattern, and joint stiffness. Where appropriate, pooled standardized mean differences (SMD) were calculated using random-effects models. Certainty of evidence was evaluated using the GRADE approach. **Results**: Nineteen studies (16 OA, 3 TKR; *n* = 7369 participants) were included, with eleven studies eligible for meta-analysis. OA individuals with unstable knees demonstrated significantly lower limb muscle strength compared with stable counterparts (SMD = −0.49, 95% CI −0.81 to −0.16, *p* = 0.003). Muscle co-contraction did not differ significantly between groups (SMD = 0.12, 95% CI −0.70 to 0.94, *p* = 0.77). The overall certainty of evidence was rated as very low. **Conclusions**: Knee instability in OA populations is associated with reduced lower limb muscle strength, although evidence quality is limited and findings regarding neuromuscular control strategies remain inconclusive. Evidence in TKR populations is scarce. Future studies should investigate muscle activation patterns and dynamic joint stabilization during functional tasks to clarify the neuromuscular mechanisms underlying knee instability.

## 1. Introduction

Knee instability is highly prevalent among individuals with knee osteoarthritis (OA), with up to 72% of patients reporting sensations of the knee buckling or giving way [1]. This perception may persist in approximately one-third of patients following total knee replacement (TKR) [2,3] and is recognized as a leading cause of postoperative dissatisfaction and revision surgery [3,4]. Knee instability is likely multifactorial, although causal mechanisms remain incompletely understood [5,6,7]. Proposed contributors include joint laxity, pain, muscle weakness, proprioceptive deficits, and altered neuromuscular control strategies [5,6,7,8,9]. In TKR, instability may additionally arise from implant design, surgical technique, component alignment, or patient-specific factors [10,11].

A joint is considered stable when it can sustain an appropriate functional position throughout its entire range [11]. Knee stability depends on both passive (intrinsic) and active (extrinsic) mechanisms [11,12,13,14]. Passive structures, including ligaments, menisci, capsule, and articular geometry, provide mechanical restraint and joint congruity [12,14]. Damage or insufficiency of these structures can lead to instability and abnormal joint loading, increasing the risk of OA development and progression [15]. In TKR, where the anterior cruciate ligament is frequently sacrificed and meniscal structures are absent, implant geometry and soft-tissue balancing play a critical role in maintaining stability [11]. Malalignment or inadequate balancing may alter tibiofemoral mechanics and contribute to perceived instability [16].

Active stabilization is provided primarily by the surrounding musculature [11]. The quadriceps and hamstring muscle groups generate compressive forces across the joint and contribute to the control of tibiofemoral translation [12,17]. Both muscle strength and coordinated activation are essential for maintaining stability and effectiveness during functional activities [18,19,20]. Individuals with knee OA commonly exhibit reduced quadriceps and hamstring strength, impaired activation, and altered neuromuscular control [21,22,23]. Similar deficits often persist following TKR, including muscle weakness, activation failure, and prolonged co-contraction patterns that may contribute to a stiff-knee gait strategy [24,25,26,27].

Despite the recognized role of muscles in joint stabilization, most research addressing knee instability, particularly in TKR populations, has focused predominantly on mechanical or surgical factors [3,4,10,28,29,30]. Furthermore, although neuromuscular impairments are well documented in OA and TKR, fewer studies have specifically examined whether these deficits differ between individuals with stable and unstable knees. Clarifying this relationship is crucial for enhancing diagnostic approaches and informing rehabilitation strategies that target instability.

In the literature, knee instability has been defined using both subjective and objective assessment methods. Subjective measures usually include self-reported feelings of buckling, giving way [7,8], or items from instability-related questionnaires [2,6], while objective measures involve assessments of mechanical laxity and instrumented joint testing. Due to this variation in classification approaches, the current review considered study-specific definitions of stable and unstable knees and explored their relationships with specific neuromuscular parameters, such as muscle strength, muscle power, joint stiffness, and muscle activation patterns.

Therefore, the purpose of this systematic review and meta-analysis was to synthesize the available evidence regarding neuromuscular differences between stable and unstable knees in individuals with OA and TKR. Specifically, we aimed to address the following questions:

- How have knee instability and neuromuscular parameters (muscle strength, muscle power, joint stiffness, and muscle activation patterns) been assessed?
- Is lower-limb muscle strength associated with knee instability?
- Is muscle activity associated with knee instability?
- Is joint stiffness associated with knee instability?

A better understanding of these relationships may help inform future strategies for assessing, preventing, and managing knee instability.

## 2. Materials and Methods

### 2.1. Literature Search and Study Selection

This systematic review and meta-analysis were conducted in accordance with the Preferred Reporting Items for Systematic Reviews and Meta-Analysis (PRISMA 2020) guidelines [31], and the completed checklist is provided in Supplementary Materials. The protocol was registered on the Open Science Framework (OSF) (DOI: 10.17605/OSF.IO/94UEC). After the protocol was established, a comprehensive literature search was performed in PubMed, CENTRAL, Scopus, and EMBASE from database inception to 3 May 2022 and updated on 20 January 2025. Google Scholar was also screened to identify additional references (first 100 results). The search strategy combined terms related to knee osteoarthritis or total knee replacement, instability, and neuromuscular function. The full list of keywords is provided in the Supplementary Materials.

Titles and abstracts were screened independently by two reviewers (A.P.L., L.C.), followed by a full-text review of potentially eligible studies. Disagreements were resolved through discussion.

Studies were eligible if they:

- Included human participants with knee OA or TKR;
- Assessed knee instability using either subjective measures (i.e., direct questions regarding perceived instability or the instability item from the Knee Outcome Survey-Activities of Daily Living Scale) or objective measures;
- Evaluated at least one neuromuscular parameter (i.e., muscle strength, muscle power, joint stiffness, or muscle activation pattern);
- Examined the relationship between knee instability and at least one neuromuscular function (i.e., muscle strength, muscle power, joint stiffness, or muscle activation pattern).

We excluded reviews, conference abstracts, studies focused exclusively on rehabilitation intervention outcomes, ligament injury or reconstruction, revision surgery, arthrosis, or non-knee conditions. Only articles written in English or French were considered. There were no restrictions on implant type, OA severity and type (medio/lateral or patellofemoral knee OA), lower limb alignment, or the laterality of either TKR or OA. No publication year restriction was applied. Reference lists of all articles that met the eligibility criteria were searched and screened for additional articles. Studies were included in the meta-analysis when sufficient data were available to calculate standardized mean differences between stable and unstable groups.

### 2.2. Data Extraction

The primary outcome of this review was neuromuscular function, including muscle strength, muscle power, joint stiffness, and muscle activation patterns, in individuals with stable and unstable knees in the context of knee OA or TKR. Two reviewers independently extracted data using a standardized form. Extracted information included publication characteristics (author, year, journal), sample size, population type (OA or TKR), participant characteristics (age, sex distribution, OA severity, and instability status), methods used to assess instability and each neuromuscular parameter, reported differences between stable and unstable groups, and any associations between instability and neuromuscular measures. Discrepancies were resolved through discussion and consensus.

### 2.3. Data Analysis

Meta-analyses were conducted when sufficient homogeneity existed across studies in terms of population, outcomes, and assessment methods. Pooled standardized mean differences (SMDs) with corresponding 95% confidence intervals (CIs) were calculated using a random-effects model based on the DerSimonian–Laird method to account for between-study variability. The SMD was computed as the mean difference between unstable and stable groups divided by the pooled standard deviation (SD). Effect sizes were interpreted according to Cohen’s conventions (small < 0.5; moderate 0.5–0.8; large ≥ 0.8) [32]. Prediction intervals were also calculated to estimate the expected range of effects in future studies.

All statistical analyses were performed using RStudio (version 4.1.3, R Foundation for Statistical Computing, Boston, MA, USA) [33]. Statistical heterogeneity was assessed using the *I*^2^ statistic, with values ≥ 50% considered indicative of substantial heterogeneity.

When numerical data were not directly reported but were presented graphically, attempts were made to contact corresponding authors to obtain the original values. Only one study was detected, and no response was received; thus, data were extracted using a digital plot-digitizing software [34]. When studies reported multiple subgroups within the stable or unstable categories (e.g., mild instability, instability affecting function), subgroup data were combined to generate a single comparison and avoid double-counting. When multiple measurement methods were reported for the same outcome, the most commonly used method across included studies was selected to improve comparability.

To enhance methodological consistency, only studies assessing neuromuscular parameters—muscle strength, muscle power, joint stiffness, and muscle activation patterns—during tasks representative of activities of daily living and those evaluating quadriceps and/or hamstring function were included in the quantitative synthesis. Subgroup analyses comparing OA and TKR populations were not performed due to the limited number of available TKR studies.

### 2.4. Risk of Bias and Overall Quality of Evidence

Risk of bias was independently assessed by two reviewers (A.P.L., L.C.) using a modified version of the Downs and Black checklist [35], previously adapted for observational studies [36]. Because the included studies were non-interventional, items specific to randomized or interventional designs were removed. The modified scale comprised 16 items across four domains: reporting quality, external validity, internal validity, and confounding, with an overall maximum possible score of 17. Studies were categorized as having a low risk of bias (score > 75% of the maximum), moderate risk (60–74%), or high risk (score < 60%). Discrepancies between reviewers were resolved through discussion and consensus.

Potential publication bias was visually assessed using contour-enhanced funnel plots [37]. Because fewer than 10 studies were included in each meta-analysis, statistical tests for funnel plot asymmetry (e.g., Egger’s regression test) were not performed, consistent with methodological recommendations [38].

Lastly, the overall certainty of evidence for each pooled outcome was evaluated using the Grading of Recommendations Assessment, Development, and Evaluation (GRADE) approach [39]. Two reviewers (A.P.L., L.C.) independently rated the quality of evidence as high, moderate, low, or very low across the domains of study limitations (risk of bias), consistency of results, directness of evidence, and precision of estimates. Evidence was downgraded when: (1) the primary outcome included studies with high risk of bias (Downs and Black score < 60% of the maximum), (2) substantial heterogeneity was present (*I*^2^ ≥ 50%), (3) confidence intervals were wide or imprecise, and (4) publication bias was suspected or could not be assessed due to the small number of studies [40]. Disagreements were resolved through consensus.

## 3. Results

**Figure 1:**
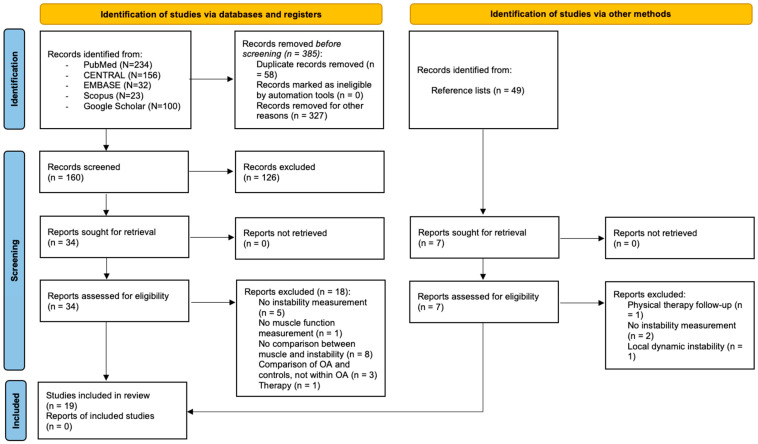
Flow diagram of the selection process (OA: Osteoarthritis).

A total of 738 records were identified through database searching (Figure 1). After screening titles and abstracts, 34 articles were assessed for full-text eligibility. Three additional studies were identified through reference list screening and were subsequently included. Nineteen studies met the inclusion criteria and were included in the systematic review. Of these, 11 [2,6,7,9,41,42,43,44,45,46,47] studies provided sufficient quantitative data (means and standard deviations for stable and unstable groups) to be included in the meta-analysis. Among the studies included in the quantitative synthesis, nine assessed muscle strength outcomes and four assessed muscle co-contraction. Studies that met the inclusion criteria but did not provide data suitable for pooling were retained for narrative synthesis and descriptively summarized in the Discussion section. A detailed summary of included studies is provided in the Supplementary Materials Table S3.

### 3.1. Study Characteristics

#### 3.1.1. OA Populations

Sixteen studies investigated individuals with knee OA, published between 2005 and 2021 (Table 1), comprising a total of 6996 participants. Unstable participants represented more than 50% of the OA cohort in seven studies, whereas in six studies the proportion was below 50% [41,42,43,44,48,49]. After excluding studies with unclear instability prevalence, 39% of individuals with OA reported knee instability. Across studies, knees were classified as stable or unstable primarily based on self-reported episodes of buckling, giving way, or instability-related questionnaire items, with detailed assessment methods described in Section 3.1.1.

The weighted mean age across studies was 65.5 years, and the proportion of female participants per study averaged 60 ± 17%. Most studies included individuals with established OA (Kellgren–Lawrence grade ≥ 2) [6,8,41,44,45,50]. One study compared early versus established OA [42], while another focused exclusively on severe OA [50]. Four cohort studies [43,48,49,51] included individuals either diagnosed with OA or at high risk of knee OA recruited from The Multicenter Osteoarthritis Study (MOST) [49,51].

**Table 1:** Summary of the demographics.

Reference	Type	No of Subjects	% of Unstable Patients	Mean Age	Gender	OA Grade
Chaudhari et al. (2019) [46]	OA	35	57%	59.9 ± 8.0	69% F	Severe
Farrokhi et al. (2015) [44]	OA	53	32%	63.0 ± 7.8 *	45% F	KL 2–4
Felson et al. (2007) [48]	OA	2351	12%	63.5	F/M	KL 0–4 25% KL ≥ 2
Fleeton et al. (2016) [2]	TKR	313	24%	65.1 ± 6.2	55% F	N/A
Freisinger et al. (2017) [50]	OA	29	Unclear	58.6 ± 7.5	66% F	KL 2–4
Gustafson et al. (2016) [41]	OA	52	33%	62.6 ± 7.2 *	46% F	KL 2–4
Hamilton et al. (2020) [52]	TKR	42	0% self-reported. Loose: 21.4% coronal and 23.8% sagittal planes.	73.0 ± 8.1	64% F	N/A
Knoop et al. (2012) [7]	OA	283	67%	61.6 ± 7.4	64% F	67% KL ≥ 2
Lewek et al. (2005) [9]	OA	21	81%	49.3 ± 7.0	33% F	Unknown
Rao et al. (2023) [53]	TKR	18	44%	68.9 ± 8.3	63% F	N/A
Sanchez-Ramirez et al. (2016) [42]	OA	33	21%	69.2 ± 5.9 *	100% F	KL 1–4 58% KL ≥ 2
Schmitt et al. (2008) [6]	OA	52	62%	61.9 *	42% F	KL 2–4
Schmitt and Rudolph (2008) [45]	OA	20	50%	64.6 *	45% F	KL 2–4
Schrijvers et al. (2021) [47]	OA	40	50%	67 ± 8 *	60% F	KL 0–4 78% KL ≥ 2
Segal et al. (2015) [51]	OA	1826	Unclear	67.4 ± 7.7	60% F	KL 0–4 43% KL ≥ 2
Shakoor et al. (2017) [49]	OA	1803	36%	67.6 ± 7.7	61% F	KL 0–4 54% KL ≥ 2
Sharma et al. (2015) [43]	OA	212	36%	64.6 ± 10.1	77% F	KL 0–4 89% KL ≥ 2
Skou et al. (2014) [8]	OA	100	76%	62.4 ± 7.3	52% F	KL 2–4
Van der Esch et al. (2006) [54]	OA	86	No self-reported instability, only laxity.	63.6 ± 9.1	76% F	KL 0–4 75% Right KL ≥ 2 73% Left KL ≥ 2

F: female, KL: Kellgren-Lawrence grade, M: male, N/A: not applicable, OA: osteoarthritis, TKR: total knee replacement. *: weighted average ± SD.

#### 3.1.2. TKR Populations

Three studies evaluated individuals following TKR, published between 2016 and 2023 (Table 1), with a total sample of 373 participants. The largest cohort was derived from the Maximum Recovery After Knee Replacement (MARKER) study [2], in which 72% of participants reported instability preoperatively and 32% remained unstable six months after surgery. A small subset of participants (*n* = 10) who developed postoperative instability was excluded from the original analysis. Additional studies included smaller samples evaluating postoperative self-reported instability [53] or joint laxity [52]. Classification of knee stability was based on postoperative self-reported instability and, in some studies, objective joint laxity assessments, as detailed in Section 3.3.1.

Across TKR studies, the weighted mean age was 66.2 years, and females represented 61 ± 5% of participants.

### 3.2. Risk of Bias

The mean score on the modified Downs and Black scale across included studies was 11.3 ± 2.0 (range: 9–16), indicating an overall moderate risk of bias (Supplementary Materials, Tables S1 and S2). None of the studies reported blinding of outcome assessors. Nine of the 19 studies adjusted for relevant confounding variables. Five studies were classified as low risk of bias, seven as moderate risk, and the remainder as high risk. Detailed scoring procedures are provided in the Supplementary Materials (pages S3–S15).

### 3.3. Neuromuscular Parameters and Assessment Methods

#### 3.3.1. Assessment of Instability and Neuromuscular Function

With the exception of two studies [8,54], knee instability was primarily assessed using self-reported measures. Nine studies evaluated the occurrence of buckling or giving-way episodes over specified recall periods (e.g., past 4 weeks [47] or 3 months [7,42,43,48,49,51,53]) or during specific activities [8]. Eight studies used an instability-related item from the Knee Outcome Survey-Activities of Daily Living Scale (KOS-ADLS) [2,6,9,41,44,45,46,50]. Two studies assessed mechanical instability using joint laxity testing, including manual stress tests (e.g., medial/lateral stress and the anterior drawer test) [52] and instrumented varus-valgus measurements [54].

Neuromuscular function was evaluated across four primary domains: muscle strength, muscle power, joint stiffness, and muscle activation patterns (Table 2). Muscle strength was most assessed using maximal voluntary isometric [2,6,8,41,42,44,45,46,48,50] or isokinetic contraction [7,42,43,49,54] of knee extensors and flexors. Isometric dynamometry was assessed with the knee either at 60° or 90° of flexion, while isokinetic was performed at 60°/s or 120°. Lower limb power was assessed through the stair climb power test [2] and a leg extensor power rig [52]. Stiffness was primarily self-reported through Western Ontario and McMaster Universities Osteoarthritis Index (WOMAC) [41,44,54]; however, one study calculated it according to the knee joint moment and angle in the sagittal plane [41]. Electromyography was used to quantify muscle activation, including measures of activation amplitude [47,53], muscle synergy [53], co-activation [9,42,45], and co-contraction indices [47]. Detailed descriptions of outcome measures are provided in the Supplementary Materials (Tables S3–S5).

#### 3.3.2. Is Muscle Strength Related to Knee Instability in OA Patients?

**Figure 2:**
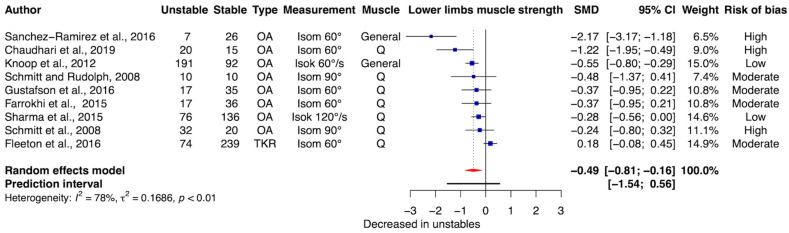
Standardized Mean Difference (SMD) and 95% Confidence Interval (CI) for the lower limb muscle strength. The black horizontal line represents the 95% prediction interval. The vertical solid line at 0 represents no difference. Negative values represent lower values in the unstable group compared to the stable one. OA: osteoarthritis, TKR: total knee replacement, Isom: isometric, Isok: isokinetic, General: general muscle strength, Q: quadriceps muscle. Random effect model SMD = −0.49 [−0.81; −0.16], *p* = 0.0034. [2,6,7,41,42,43,44,45,46].

Meta-analysis demonstrated significantly reduced lower-limb muscle strength in individuals with unstable knees compared with stable knees (SMD = −0.49, 95% CI [−0.81, −0.16], *p* = 0.0034) (Figure 2), representing a moderate effect size. Between-study heterogeneity was substantial (τ^2^ = 0.17, 95% CI [0.08, 1.20]; *I*^2^ = 78%, 95% CI [59, 89%]). The prediction interval ranged from −1.54 to 0.56, indicating variability in the magnitude and direction of effects across studies.

A sensitivity analysis excluding the study involving TKR participants [2] yielded a larger pooled effect size favoring reduced strength in unstable OA knees (SMD = −0.57, 95% CI [−0.87, −0.28], *p* = 0.0001), with moderate heterogeneity (τ^2^ = 0.09, 95% CI [0.04, 1.14]; *I*^2^ = 62%, 95% CI [17, 82%]). The prediction interval ranged from −1.41 to 0.26.

**Figure 3:**
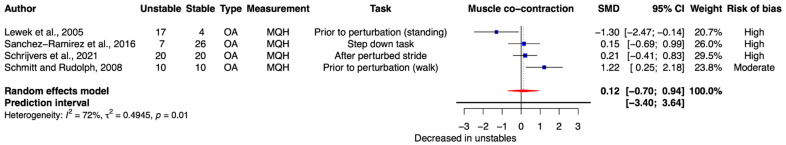
Standardized Mean Difference (SMD) and 95% Confidence Interval (CI) of the medial thigh muscle co-contraction. The black horizontal line represents the 95% prediction interval. The vertical solid line at 0 represents no difference. Negative values represent lower values in the unstable group compared to the stable one. OA: osteoarthritis, MQH: medial quadriceps and hamstring muscles. Random effect model SMD = 0.12 [−0.70;0.94], *p* = 0.7717 [9,42,45,47].

Meta-analysis of muscle co-contraction outcomes showed no significant differences between unstable and stable OA groups (SMD = 0.12, 95% CI [−0.70, 0.94], *p* = 0.77) (Figure 3). Substantial heterogeneity was observed between studies (τ^2^ = 0.49, 95% CI [0.07, 12.0]; *I*^2^ = 72%, CI [20, 90%]). The prediction interval ranged from −3.40 to 3.54, indicating considerable uncertainty regarding the direction and magnitude of effects. No studies involving TKR populations provided sufficient data for inclusion in this analysis.

#### 3.3.3. Is Knee Stiffness Related to Knee Instability in OA Patients?

Only two studies examined the relationship between joint stiffness and knee instability in individuals with OA. Due to the limited number of studies and methodological heterogeneity, a quantitative synthesis was not performed.

### 3.4. Publication Bias

**Figure 4:**
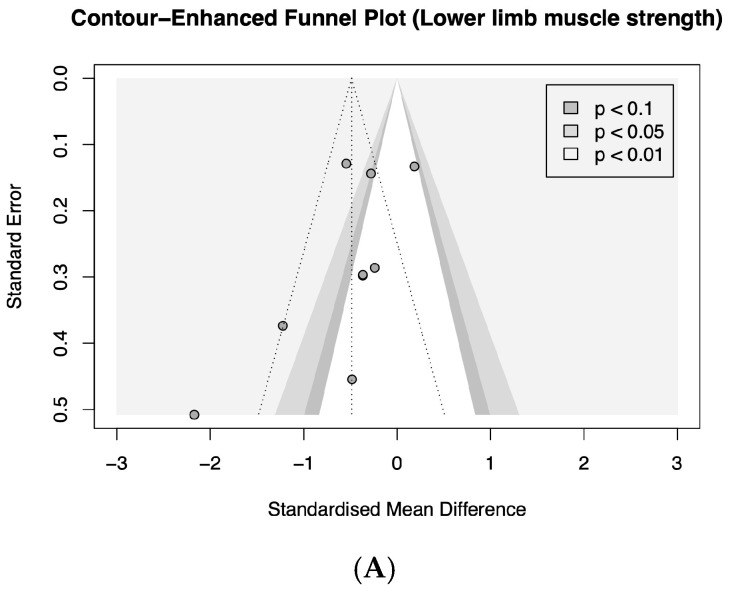

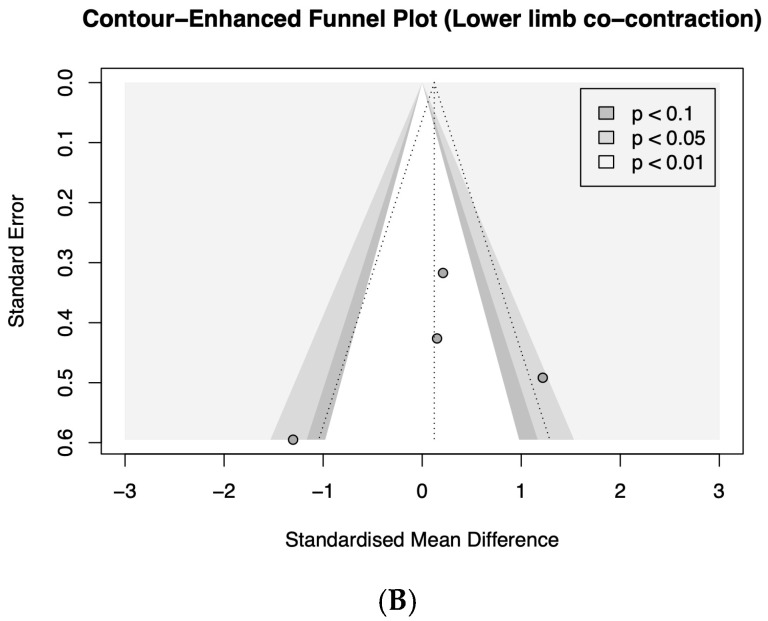
Contour-enhanced funnel plot for lower limb muscle strength (**A**) and co-contraction (**B**). The vertical dashed line represents the random effect model standardized mean difference. The two dashed diagonal lines represent the region within which 95% of the studies are expected to lie in absence of bias and heterogeneity. The contour-enhanced funnel plot displays areas of statistical significance of the effect sizes.

Visual inspection of contour-enhanced funnel plots suggested asymmetry for both muscle strength (Figure 4A) and co-contraction outcomes (Figure 4B), raising the possibility of publication bias. Formal statistical tests for funnel plot asymmetry were not conducted due to the small number of included studies (<10), consistent with methodological recommendations.

### 3.5. Quality of Evidence

The certainty of evidence for all meta-analysis outcomes was rated as very low according to the GRADE framework (Table 3). Evidence was downgraded primarily due to high heterogeneity, methodological limitations, and inability to reliably assess publication bias. For muscle co-contraction, additional downgrading occurred due to the risk of bias and imprecision of effect estimates. No factors justified upgrading the quality of evidence. These ratings indicate that the true effect is likely to differ substantially from the estimated effect sizes [41].

## 4. Discussion

This systematic review and meta-analysis summarized the available evidence regarding neuromuscular differences between individuals with stable and unstable knees in the context of knee OA and TKR. The primary finding was that individuals with unstable OA demonstrated significantly reduced lower-limb muscle strength compared with those without instability. In contrast, muscle co-contraction patterns did not differ significantly between groups, although substantial heterogeneity was observed. Evidence regarding other neuromuscular domains, including joint stiffness, muscle power, and muscle activation patterns, remains limited, and data specific to unstable TKR populations are scarce. To our knowledge, this is the first review to comprehensively evaluate neuromuscular function in relation to knee instability across both OA and TKR populations.

Neuromuscular impairments in knee OA are well documented [55,56] and include quadriceps and hamstring weakness [57,58], altered muscle activation patterns, impaired ability to control quadriceps force [20,59], and reduced knee proprioception [60]. However, comparatively little attention has been directed toward distinguishing and characterizing neuromuscular characteristics between individuals who do and do not report instability. One challenge in this area is the lack of consensus regarding the definition and measurement of knee instability [61]. Studies frequently use mechanical laxity and perceived instability interchangeably, despite evidence suggesting these constructs represent distinct phenomena. In the present review, both subjective (i.e., self-reported instability) and objective (i.e., laxity) measures were considered to provide a broader understanding of instability-related neuromuscular deficits. Perceived instability is highly prevalent in knee OA [5,62] and may substantially influence functional performance and activity participation. Potential contributors include, but are not limited to, structural joint damage, ligamentous and/or capsule laxity, muscle weakness, and proprioceptive deficits [5,7,48]. In OA, increased varus-valgus laxity, sometimes referred to as pseudolaxity, may arise from bone erosion, joint space narrowing, and altered ligament attachment geometry [63,64]. Nevertheless, several studies have reported weak or absent associations between mechanical laxity and perceived instability in OA [6,7,46,50], whereas stronger relationships have been observed in TKR populations [65,66]. Importantly, not all TKR individuals with lax knees report instability, suggesting that neuromuscular compensatory strategies may modulate symptom perception [52].

Muscle strength emerged as the most consistently associated neuromuscular factor with instability, a finding that is in line with prior literature and current biomechanical understanding. Quadriceps strength is particularly important for knee stabilization during weight-bearing activities [56,67], and deficits in knee extensor strength are known to contribute to both OA onset and progression [57,68]. The present meta-analysis quantitatively confirmed moderate reductions in strength in unstable OA compared with stable OA, thereby strengthening the existing body of evidence. Although the certainty of evidence was very low due to heterogeneity and potential publication bias, these findings are supported by multiple observational studies reporting associations between greater quadriceps strength and lower odds of perceived instability [7,42,46]. Longitudinal evidence further suggests that individuals with greater strength may be less likely to develop or worsen instability over time [49]. Although the pain-related cycle of reduced joint use and subsequent muscle weakness is well established in OA, the directionality of the relationship between perceived instability and muscle weakness remains difficult to disentangle. Specifically, muscle weakness may contribute to instability, while instability-related pain and avoidance of weight-bearing activities may further exacerbate weakness over time. Differences in strength testing protocols across studies, particularly testing angles that do not replicate functional positions associated with instability [2], may also contribute to variability in findings.

Several established mechanisms may explain the observed association between muscle weakness and instability. Arthrogenic muscle inhibition (AMI), driven by altered afferent signaling from inflamed or damaged joint tissues, can reduce voluntary activation and promote muscle atrophy [69,70]. Pain may further exacerbate weakness by limiting activity and reinforcing the well-recognized cycle of inactivity and functional decline [71,72]. Inflammatory processes and altered neuromuscular excitability have also been linked to reduced knee extensor strength [73]. These interacting mechanisms likely contribute to impaired dynamic stabilization capacity and increased perception of instability during functional tasks.

Interventional studies provide indirect support for the clinical relevance of strength deficits. Strengthening programs have been shown to improve pain, function, and perceived instability in individuals with OA [74,75]. However, the additional benefit of targeted stabilization exercises remains uncertain for those with perceived instability [7]. In TKR populations, postoperative weakness is substantial [2] and may persist for months or years due to atrophy [27], neural inhibition [76], and AMI [69]. Limited evidence suggests that rehabilitation focusing on quadriceps and hamstring strengthening may reduce instability symptoms and potentially decrease the need for revision surgery, highlighting the importance of neuromuscular rehabilitation in this population [77].

Despite theoretical expectations that individuals with instability would exhibit increased muscle co-contraction to compensate for reduced passive stability, the present meta-analysis did not identify significant differences between stable and unstable OA groups. Findings across individual studies were inconsistent [45,47,51], likely reflecting differences in tasks, measurement methods, and instability definitions. Some evidence suggests that unstable individuals may increase medial co-contraction during challenging or perturbed tasks [45], indicating a context-dependent compensatory strategy rather than a consistent baseline characteristic. Moreover, increased co-contraction does not necessarily prevent instability symptoms [9] and may instead reflect inefficient neuromuscular control or attempts to stiffen the joint in response to perceived threat [55]. Comparisons with healthy populations consistently demonstrate greater co-contraction in OA overall [78,79], supporting the concept of a compensatory stabilization strategy, but differentiation based on instability status remains unclear.

Joint stiffness represents another potential knee stabilization mechanism [9,47] but remains poorly studied in relation to instability. Although OA populations generally demonstrate increased walking stiffness compared with healthy controls [78,80,81], limited evidence [41] suggests that individuals with unstable OA may exhibit reduced dynamic stiffness, potentially reflecting an inability to generate sufficient active stabilization during gait. Interpretation is complicated by methodological differences and the multifactorial nature of stiffness [41], which depends on both passive tissue properties and neuromuscular activation. Importantly, functional measures of dynamic stiffness may provide more objective insight into instability than self-reported measures, warranting further investigation.

Evidence regarding neuromuscular function in unstable TKR populations is particularly limited. Only one study included in the meta-analysis evaluated strength differences, and very few studies compared neuromuscular characteristics between stable and unstable TKR knees. Postoperative adaptations, including higher dynamic knee stiffness and increased quadriceps activity without a concomitant increase in the hamstrings, have been reported, but their relationship to instability perception remains unclear [79]. Preliminary findings from our laboratory identified higher values of dynamic joint stiffness for unstable TKR subjects, emphasizing the need for comparisons on stiffness and stability status in this population [82].

Muscle power also appears relevant to instability but could not be quantitatively synthesized due to limited data. Early postoperative reductions in stair-climbing power have been associated with retained instability following TKR [2], suggesting that tasks requiring rapid force generation may better reveal functional instability than isolated strength tests. Unlike maximal strength, muscle power reflects the ability to generate force quickly, which may be particularly important during dynamic activities such as stair negotiation, gait initiation, or recovery from perturbations, situations where instability symptoms are commonly reported. The limited number of studies and the heterogeneity of measurement methods, including differences in functional tasks and measurement tools, likely contributed to the inconsistent findings. Future studies should further investigate whether deficits in lower-limb power represent an independent contributor to perceived instability and whether power-targeted rehabilitation strategies may provide additional benefits beyond conventional strengthening approaches.

### 4.1. Limitations

Although this review provides novel insights into neuromuscular function in individuals with knee OA and TKR who report instability, several limitations should be considered. First, only three studies included TKR populations, and substantial variability existed in experimental protocols, outcome measures, and definitions of instability, limiting the ability to draw robust conclusions for this subgroup. Second, many included studies had relatively small sample sizes, with more than half enrolling fewer than 60 participants, which may have reduced statistical power and increased the risk of imprecision in pooled estimates.

Some cohort studies included participants based on risk factors for OA rather than confirmed disease status, meaning that statistical analyses were sometimes performed on mixed populations that included individuals without OA [48,49,51]. Additionally, not all studies directly compared stable and unstable groups; instead, some examined associations between instability and neuromuscular variables using regression approaches within a single cohort [8,54]. These methodological differences contribute to heterogeneity and complicate the interpretation of between-group effects.

The generalizability of the findings is further limited by the substantial heterogeneity across studies, the small number of eligible investigations for several outcomes, and the overall very low certainty of evidence according to GRADE criteria. The limited number of studies also precluded a formal quantitative assessment of publication bias. Finally, the functional tasks used across studies varied considerably, and some laboratory activities may not adequately represent activities of daily living, which could influence neuromuscular responses and perceptions of instability.

### 4.2. Perspectives and Future Directions

Future research should prioritize standardized, functionally relevant assessment protocols that are sensitive to instability-related symptoms while reflecting real-world functional demands. Tasks such as stair descent and gait-based assessments may be more appropriate than isolated step-down paradigms, as they require greater eccentric muscle control and rapid joint stabilization under higher loads. Experimental conditions should balance task challenge and ecological validity to better characterize the neuromuscular responses associated with daily activities and to support the development of clinically meaningful assessment tools.

From a clinical perspective, future studies should focus on identifying objective neuromuscular markers of instability, such as dynamic joint stiffness, muscle activation patterns, and co-contraction during gait, that may improve screening and patient stratification in both OA and TKR populations. Future work should also examine how these measures interact with the joint range of motion and frontal plane alignment (e.g., varus/valgus angles), particularly in later stages of OA, where stiffness and mechanical alignment may influence perceived instability. Such measures may help clinicians distinguish individuals whose instability is primarily driven by strength deficits, impaired motor control, restricted mobility, or mechanical factors, thereby supporting more targeted and individualized rehabilitation strategies.

In TKR populations, where evidence remains limited, prospective studies are needed to determine whether these neuromuscular markers can predict persistent postoperative instability and guide early intervention. Novel rehabilitation approaches should evaluate whether task-specific neuromuscular training, perturbation-based exercises, proprioceptive retraining, and power-focused strengthening provide benefits beyond conventional strengthening programs. Future work should prioritize the clinical evaluation of innovative, individualized rehabilitation strategies and determine which patients are most likely to benefit from specific neuromuscular interventions, thereby facilitating precision rehabilitation approaches for instability management.

## 5. Conclusions

This systematic review and meta-analysis aimed to clarify neuromuscular characteristics associated with stable and unstable knees in individuals with OA and TKR. The available evidence suggests that unstable OA knees are characterized by significantly reduced lower-limb muscle strength, whereas findings related to muscle co-contraction and dynamic stiffness remain inconclusive. Evidence in TKR populations is particularly limited, with only three eligible studies identified, preventing definitive conclusions.

Overall, the certainty of evidence was rated as very low, highlighting the need for high-quality research using standardized and clinically meaningful methodologies. Future investigations should focus on functionally relevant assessment and rehabilitation approaches, particularly gait-based and task-specific neuromuscular evaluations, to better characterize instability-related deficits and identify targeted intervention strategies. Such advances may help inform individualized rehabilitation protocols aimed at reducing instability and enhancing functional outcomes in individuals with knee OA and TKR.

## Figures and Tables

**Table 2:** Summary of study parameters.

Reference	Stability Measurement		Neuromuscular Function Assessment						
	Self-Reported	Laxity	Muscle Strength		Power	Stiffness		Activity	
			Isometric	Isokinetic		Measured	Self-Reported	Co-Contraction	Muscle Activity
Chaudhari et al. [46]	x	x (intra-op)	x						
Farrokhi et al. [44]	x		x				x		
Felson et al. [48]	x		x						
Fleeton et al. [2]	x		x		x				
Freisinger et al. [50]	x	x (intra-op)	x						
Gustafson et al. [41]	x	x	x			x	x		
Hamilton et al. [52]		x			x				
Knoop et al. [7]	x	x		x					
Lewek et al. [9]	x	x						x	
Rao et al. (2023) [53]	x							co-activation	activation and muscle synergy
Sanchez-Ramirez et al. [42]	x		x	x				x	x
Schmitt et al. [6]	x	x	x						
Schmitt and Rudolph [45]	x	x	x					x	
Schrijvers et al. [47]	x							x	x
Segal et al. [51]	x							co-activation	
Shakoor et al. [49]	x			x					
Sharma et al. [43]	x			x					
Skou et al. [8]	x		x						
Van der Esch et al. [54]		x		x			x		

Intra-op: intra-operation.

**Table 3:** Summary of the body of evidence using GRADE’s approach.

Outcome	SMD [95% CI]	Study Design	Sample Size	Downs and Black Scale Score	Heterogeneity	Level of Evidence (GRADE)
Muscle strength						
OA + TKR	−0.49 [−0.81; −0.16]	9× observational	444 unstable 609 stable	11.2 ± 1.4 points	*I*^2^ = 78%	⊕OOO Very Low ^2,4^
Only OA	−0.57 [−0.87; −0.28]	8× observational	370 unstable 370 stable	11.1 ± 1.5 points	*I*^2^ = 62%	⊕OOO Very Low ^2,4^
Co-contraction						
Only OA	0.12 [−0.70; 0.94]	4× observational	54 unstable 60 stable	10.0 ± 0.8 points	*I*^2^ = 72%	⊕OOO Very Low ^1,2,3,4^

CI: confidence interval; GRADE: grades of recommendation, assessment, development, and evaluation; OA: osteoarthritis; SMD: standardized mean difference. “⊕OOO Very Low” quality: very little confidence in the effect estimate. ^1^: downgraded for risk of bias (mean score of included studies was less than 10.2). ^2^: downgraded for inconsistency (results were heterogeneous across the included studies, *I*^2^ ≥ 50%). ^3^: downgraded for imprecision (the 95% CI covers both effect sides, “decreased and increased in unstable patients”). ^4^: downgraded for publication bias (unable to determine because less than 10 studies were included).

## Data Availability

The original contributions presented in this study are included in the article/Supplementary Materials. Further inquiries can be directed to the corresponding authors.

## Supplementary Materials

The following supporting information can be downloaded at: https://www.mdpi.com/article/10.3390/clinpract16040074/s1: PRISMA 2020 checklist, Table S1: Summary of the risk of bias assessment using the modified Downs and Black scale [36]. Table S2: Risk of bias assessment results per study. Table S3: Summary of studies included in the systematic review. Table S4: Summary of included studies in the lower limbs muscle strength meta-analysis. Table S5: Summary of included studies in the lower limbs muscle co-contraction meta-analysis.

## Abbreviations

The following abbreviations are used in this manuscript:

OA	Osteoarthritis
TKR	Total Knee Replacement
SMD	Standardized Mean Differences
AMI	Arthrogenic Muscle Inhibition
