# Supplementary material for: Neuromuscular Characteristics Associated with Knee Instability in Osteoarthritis and After Total Knee Replacement: A Systematic Review and Meta-Analysis
Source: Clin Pract. 2026 Apr 14;16(4):74. doi: 10.3390/clinpract16040074 (PMC13114445; doi:10.3390/clinpract16040074)
Supplement: Supplementary file 1 [file clinpract-16-00074-s001.zip › clinpract-4224429-supplementary.pdf]

Supplementary material

Table of Contents

Literature search keywords 2

Risk of bias assessment of included studies 3

Summary of studies included in the systematic review 20

References 29

List of tables

Table S1. Summary of the risk of bias assessment using the modified Downs and Black scale (Munn et al., 2010). 3

Table S2. Risk of bias assessment results per study. 4

Table S3. Summary of studies included in the systematic review 20

Table S4. Summary of included studies in the lower limbs muscle strength meta-analysis.. 27

Table S5. Summary of included studies in the lower limbs muscle co-contraction meta-analysis........ 27

This supplementary material has been provided by the authors to give readers additional information regarding their work.

Literature search keywords

The following keywords were used in each database.

PubMed

7	#6 NOT #4
6	#1 AND #5
5	#2 AND #3
4	"Arthritis, Rheumatoid"[Mesh] OR "Animals, Laboratory"[Mesh] OR "Proprioception"[Mesh] OR hip OR "Amputation"[Mesh] OR "Cadaver"[Mesh] OR tumor
3	muscle OR "Muscle Contraction"[Mesh] OR "Muscle Weakness"[Mesh] OR "Muscle Strength"[Mesh] OR "Electromyography"[Mesh] OR co-contraction OR muscle recruitment OR muscle weakness OR muscle power OR neuromuscular
2	"Joint Instability"[Mesh] OR instability OR stability OR laxity
1	"Osteoarthritis, Knee"[Mesh] OR "Arthroplasty, Replacement, Knee"[Mesh]

CENTRAL, Scopus, EMBASE, Google Scholar

(Knee Osteoarthritis OR Knee replacement) AND (Joint Instability OR instability OR stability OR laxity) AND (muscle OR Muscle Contraction OR Muscle Weakness OR Muscle Strength OR Electromyography OR co-contraction OR muscle recruitment OR muscle weakness OR muscle power OR neuromuscular) NOT (Rheumatoid Arthritis OR Animals OR proprioception OR hip OR amputation OR cadaver OR tumor)

Risk of bias assessment of included studies

Two reviewers (AL, LC) evaluated the risk of bias using an adapted version of the Downs and Black scale.[1] This checklist has 16 items (reporting [1-3, 5-7, 10], external validity [11,12], internal validity (bias) [15, 16, 18, 20], and internal validity (confounding) [21,22, 25]) from the original Downs and Black scale.[2] The minimum score is 0 and the maximum is 17. The overall risk of bias is high if the total is <60% (*i.e.*, <10.2), moderate if 60-74%, and low if >75% (*i.e.*, >12.75).

Regarding **item 5**, we are considering all potential confounders; however, if the primary described confounders are BMI or walking speed, we state this as "partially". When at least age and sex confounders are used, we rate it as "Yes". Regarding **item 11**, papers must specify the source of patients and the inclusion and exclusion criteria. If the study did not report the proportion of the source population from which the patients are derived, the question is answered as "unable to determine". Regarding **item 21**, authors must provide the place from which the patients were recruited. Then, we consider this item as "Yes" if the groups of interest (unstable and stable) are drawn from the same sample. This means that the authors built subgroups from the main sample.

The results of the risk of bias assessment are presented in Table S1. A precise description of the assessment per study, and justifications are given in Table S2.

Table S1. Summary of the risk of bias assessment using the modified Downs and Black scale (Munn et al., 2010).

Reference	Reporting	External validity	Internal validity - bias	Internal validity - confounding	TOTAL	Overall risk of bias
Chaudhari et al., 2019	6	0	3	1	**10**	*High*
Farrokhi et al., 2015	7	0	3	1	**11**	*Moderate*
Felson et al., 2007	5	1	3	3	**13**	*Low*
Fleeton et al., 2016	5	1	3	2	**12**	*Moderate*
Freisinger et al., 2017a	6	0	3	1	**11**	*Moderate*
Gustafson et al., 2016	8	0	3	1	**12**	*Moderate*
Hamilton et al., 2020	5	0	3	2	**11**	*Moderate*
Knoop et al., 2012	8	0	3	2	**13**	*Low*
Lewek et al., 2005	6	0	3	0	**9**	*High*
Rao et al., 2023	6	0	3	0	**9**	*High*

Sanchez-Ramirez et al., 2016	6	0	3	1	**10**	*High*
Schmitt and Rudolph, 2008	6	0	3	2	**11**	*Moderate*
Schmitt et al., 2008	6	0	3	0	**9**	*High*
Schrijvers et al., 2021	6	0	3	1	**10**	*High*
Segal et al., 2015	7	1	3	3	**15**	*Low*
Shakoor et al., 2017	8	1	3	3	**16**	*Low*
Sharma et al., 2015	8	0	3	2	**13**	*Low*
Skou et al., 2014	7	0	3	1	**11**	*Moderate*
Van der Esch et al., 2006	6	0	3	0	**9**	*High*
**Mean**	**6.4**	**0.2**	**3.0**	**1.4**	**11.3**	
**SD**	**1.0**	**0.4**	**0.0**	**1.0**	**2.0**	

Table S2. Risk of bias assessment results per study.

**Reference**	**Chaudhari et al., 2019**			**Notes, justification**
**REPORTING**	**0**	**1**	**2**	
1. Is the hypothesis/aim/objective of the study clearly described?		Yes		
2. Are the main outcomes to be measured clearly described in the introduction or methods?<br>*If the main outcomes are first mentioned in the results section, the question should be answered no.*		Yes		
3. Are the characteristics of the patients included in the study clearly described?<br>*In cohort studies and trials, inclusion and/or exclusion criteria should be given. In case control studies, a case-definition and the source of controls should be given.*		Yes		
5. Are the distribution of principal confounders in each group of subjects to be compared clearly described?<br>*A list of principal confounders is provided.*	No			No list of confounders is given.
6. Are the main findings of the study clearly described?<br>*Simple outcome data should be reported for all major findings so that the reader can check the major analyses and conclusions.*		Yes		
7. Does the study provide estimates of the random variability in the data for the main outcomes?<br>*In non-normally distributed data, the inter-quartile range of results should be reported. In normally distributed data the SE, SD or CI should be reported. If the distribution of the data is not described, it must be assumed that the estimates used were appropriate and the question should be answered yes.*		Yes		
10. Have actual probability values been reported (*e.g. 0.035 rather than <0.05*) for the main outcomes except where the probability value is less than 0.001?		Yes		
**EXTERNAL VALIDITY**				
11. Were the subjects asked to participate in the study representative of the entire population from which they were recruited?<br>*The study must identify the source population for patients and describe how the patients were selected. Patients would be representative if they comprised the entire source population, an unselected sample of consecutive patients, or a random sample. Random sampling is only feasible where a list of all members of the relevant. population exists. Where a study does not report the proportion of the source population from which the patients are derived, the question should be answered as unable to determine.*	Unable to determine			No mention of the initial total size of population before applying the inclusion and exclusion criteria.
12. Were those subjects who were prepared to participate representative of the entire population from which they were recruited?<br>*The proportion of those asked who agreed should be stated. Validation that the sample was representative would include demonstrating that the distribution of the main confounding factors was the same in the study sample and the source population.*	Unable to determine			No mention of the proportion of those who agreed to participate. If so, no mention of the proportion of the participants that were excluded after data collection.
**INTERNAL VALIDITY – BIAS**				
15. Was an attempt made to blind those measuring the main outcomes of the intervention?	No			No blinding.
16. If any of the results of the study were based on “data dredging”, was this made clear?<br>*Any analyses that had not been planned at the outset of the study should be clearly indicated. If no retrospective unplanned subgroup analyses were reported, then answer yes.*		Yes		

18. Were the statistical tests used to assess the main outcomes appropriate?<br>*The statistical techniques used must be appropriate to the data. For example, non-parametric methods should be used for small sample sizes. Where little statistical analysis has been undertaken but where there is no evidence of bias, the question should be answered yes. If the distribution of the data is not described, it must be assumed that the estimates used were appropriate and the question should be answered yes.*		Yes		
20. Were the main outcome measures used accurate (valid and reliable)?<br>*For studies where the outcome measures are clearly described, the question should be answered yes. For studies which refer to other work or that demonstrates the outcome measures are accurate, the question should be answered as yes.*		Yes		
**INTERNAL VALIDITY – CONFOUNDING (SELECTION BIAS)**				
21. Were the patients in different intervention groups (trials and cohort studies) or were the cases and controls (case-control studies) recruited from the same population?<br>*For example, patients for all comparison groups should be selected from the same hospital. The question should be answered unable to determine for cohort and case-control studies where there is no information concerning the source of patients included in the study.*		Yes		
22. Were study subjects in different intervention groups (trials and cohort studies) or were the cases and controls (case-control studies) recruited over the same period of time?<br>*For a study which does not specify the time period over which patients were recruited, the question should be answered as unable to determine.*	Unable to determine			Unable to determine the timeline of the recruitment.
25. Was there adequate adjustment for confounding in the analyses from which the main findings were drawn?<br>*This question should be answered no for trials if: the main conclusions of the study were based on analyses of treatment rather than intention to treat; the distribution of known confounders in the different treatment groups was not described; or the distribution of known confounders differed between the treatment groups but was not taken into account in the analyses. In non-randomized studies if the effect of the main confounders was not investigated or con- founding was demonstrated but no adjustment was made in the final analyses the question should be answered as no.*	No			No adjustment for the confounders in the analyses.

**Reference**	**Farrokhi et al., 2015**			**Notes, justification**
**REPORTING**	**0**	**1**	**2**	
1. Is the hypothesis/aim/objective of the study clearly described?		Yes		
2. Are the main outcomes to be measured clearly described in the introduction or methods?		Yes		
3. Are the characteristics of the patients included in the study clearly described?		Yes		
5. Are the distribution of principal confounders in each group of subjects to be compared clearly described?		Partially		Confounders are age, sex, height, BMI, radiographic disease severity, gait speed.
6. Are the main findings of the study clearly described?		Yes		
7. Does the study provide estimates of the random variability in the data for the main outcomes?		Yes		
10. Have actual probability values been reported (*e.g. 0.035 rather than <0.05*) for the main outcomes except where the probability value is less than 0.001?		Yes		
**EXTERNAL VALIDITY**				
11. Were the subjects asked to participate in the study representative of the entire population from which they were recruited?	No			Source of population was not mentioned, neither the initial population size.
12. Were those subjects who were prepared to participate representative of the entire population from which they were recruited?	Unable to determine			No mention of the proportion of those who agreed to participate. If so, no mention of the proportion of the participants that were excluded after data collection.
**INTERNAL VALIDITY – BIAS**				
15. Was an attempt made to blind those measuring the main outcomes of the intervention?	No			No blinding.
16. If any of the results of the study were based on "data dredging", was this made clear?		Yes		

18. Were the statistical tests used to assess the main outcomes appropriate?		Yes		
20. Were the main outcome measures used accurate (valid and reliable)?		Yes		
**INTERNAL VALIDITY – CONFOUNDING (SELECTION BIAS)**				
21. Were the patients in different intervention groups (trials and cohort studies) or were the cases and controls (case-control studies) recruited from the same population?	Unable to determine			The source of patients is not provided.
22. Were study subjects in different intervention groups (trials and cohort studies) or were the cases and controls (case-control studies) recruited over the same period of time?	Unable to determine			Unable to determine the timeline of the recruitment.
25. Was there adequate adjustment for confounding in the analyses from which the main findings were drawn?		Yes		

**Reference**	**Felson et al., 2007**			**Notes, justification**
**REPORTING**	**0**	**1**	**2**	
1. Is the hypothesis/aim/objective of the study clearly described?	No			Authors described what they did, the aim/hypothesis/objective is not clearly described. The objective is only described in the abstract.
2. Are the main outcomes to be measured clearly described in the introduction or methods?	No			Isometric quadriceps strength test is not clearly described (no mention to the knee flexion angle).
3. Are the characteristics of the patients included in the study clearly described?		Yes		
5. Are the distribution of principal confounders in each group of subjects to be compared clearly described?			Yes	The confounders are. Age, sex, BMI, quadriceps strength, knee pain severity.
6. Are the main findings of the study clearly described?		Yes		
7. Does the study provide estimates of the random variability in the data for the main outcomes?		Yes		
10. Have actual probability values been reported (*e.g. 0.035 rather than <0.05*) for the main outcomes except where the probability value is less than 0.001?	No			Did not provide the exact probability (only reported p<0.05).
**EXTERNAL VALIDITY**				
11. Were the subjects asked to participate in the study representative of the entire population from which they were recruited?		Yes		
12. Were those subjects who were prepared to participate representative of the entire population from which they were recruited?		Yes		
**INTERNAL VALIDITY – BIAS**				
15. Was an attempt made to blind those measuring the main outcomes of the intervention?	No			No blinding.
16. If any of the results of the study were based on "data dredging", was this made clear?		Yes		
18. Were the statistical tests used to assess the main outcomes appropriate?		Yes		
20. Were the main outcome measures used accurate (valid and reliable)?		Yes		
**INTERNAL VALIDITY – CONFOUNDING (SELECTION BIAS)**				
21. Were the patients in different intervention groups (trials and cohort studies) or were the cases and controls (case-control studies) recruited from the same population?		Yes		
22. Were study subjects in different intervention groups (trials and cohort studies) or were the cases and controls (case-control studies) recruited over the same period of time?		Yes		

25. Was there adequate adjustment for confounding in the analyses from which the main findings were drawn?		Yes		

Reference	Fleeton et al., 2016			Notes, justification
**REPORTING**	**0**	**1**	**2**	
1. Is the hypothesis/aim/objective of the study clearly described?		Yes		
2. Are the main outcomes to be measured clearly described in the introduction or methods?		Yes		
3. Are the characteristics of the patients included in the study clearly described?		Yes		
5. Are the distribution of principal confounders in each group of subjects to be compared clearly described?	No			No list of confounders is given.
6. Are the main findings of the study clearly described?		Yes		
7. Does the study provide estimates of the random variability in the data for the main outcomes?		Yes		
10. Have actual probability values been reported (*e.g. 0.035 rather than <0.05*) for the main outcomes except where the probability value is less than 0.001?	No			Did not provide the exact probability (only reported p<0.05).
**EXTERNAL VALIDITY**				
11. Were the subjects asked to participate in the study representative of the entire population from which they were recruited?		Yes		
12. Were those subjects who were prepared to participate representative of the entire population from which they were recruited?		Yes		
**INTERNAL VALIDITY – BIAS**				
15. Was an attempt made to blind those measuring the main outcomes of the intervention?	No			No blinding.
16. If any of the results of the study were based on "data dredging", was this made clear?		Yes		
18. Were the statistical tests used to assess the main outcomes appropriate?		Yes		
20. Were the main outcome measures used accurate (valid and reliable)?		Yes		
**INTERNAL VALIDITY – CONFOUNDING (SELECTION BIAS)**				
21. Were the patients in different intervention groups (trials and cohort studies) or were the cases and controls (case-control studies) recruited from the same population?		Yes		
22. Were study subjects in different intervention groups (trials and cohort studies) or were the cases and controls (case-control studies) recruited over the same period of time?		Yes		
25. Was there adequate adjustment for confounding in the analyses from which the main findings were drawn?	No			No adjustment for the confounders in the analyses.

Reference	Freisinger et al., 2017a			Notes, justification
**REPORTING**	**0**	**1**	**2**	
1. Is the hypothesis/aim/objective of the study clearly described?		Yes		
2. Are the main outcomes to be measured clearly described in the introduction or methods?		Yes		
3. Are the characteristics of the patients included in the study clearly described?		Yes		
5. Are the distribution of principal confounders in each group of subjects to be compared clearly described?	No			No list of confounders is given.
6. Are the main findings of the study clearly described?		Yes		
7. Does the study provide estimates of the random variability in the data for the main outcomes?		Yes		
10. Have actual probability values been reported (*e.g. 0.035 rather than <0.05*) for the main outcomes except where the probability value is less than 0.001?		Yes		

**EXTERNAL VALIDITY**				
11. Were the subjects asked to participate in the study representative of the entire population from which they were recruited?	Unable to determine			No mention of the initial total size of population before applying the inclusion and exclusion criteria
12. Were those subjects who were prepared to participate representative of the entire population from which they were recruited?		Yes		
**INTERNAL VALIDITY – BIAS**				
15. Was an attempt made to blind those measuring the main outcomes of the intervention?	No			No blinding.
16. If any of the results of the study were based on "data dredging", was this made clear?		Yes		
18. Were the statistical tests used to assess the main outcomes appropriate?		Yes		
20. Were the main outcome measures used accurate (valid and reliable)?		Yes		
**INTERNAL VALIDITY – CONFOUNDING (SELECTION BIAS)**				
21. Were the patients in different intervention groups (trials and cohort studies) or were the cases and controls (case-control studies) recruited from the same population?		Yes		
22. Were study subjects in different intervention groups (trials and cohort studies) or were the cases and controls (case-control studies) recruited over the same period of time?	Unable to determine			Unable to determine the timeline of the recruitment.
25. Was there adequate adjustment for confounding in the analyses from which the main findings were drawn?	No			No adjustment for the confounders in the analyses.

**Reference**	**Gustafson et al., 2016**			**Notes, justification**
**REPORTING**	**0**	**1**	**2**	
1. Is the hypothesis/aim/objective of the study clearly described?		Yes		
2. Are the main outcomes to be measured clearly described in the introduction or methods?		Yes		
3. Are the characteristics of the patients included in the study clearly described?		Yes		
5. Are the distribution of principal confounders in each group of subjects to be compared clearly described?			Yes	The confounders are gait speed and sex.
6. Are the main findings of the study clearly described?		Yes		
7. Does the study provide estimates of the random variability in the data for the main outcomes?		Yes		
10. Have actual probability values been reported (*e.g. 0.035 rather than* <0.05) for the main outcomes except where the probability value is less than 0.001?		Yes		
**EXTERNAL VALIDITY**				
11. Were the subjects asked to participate in the study representative of the entire population from which they were recruited?	No			Source of population was not mentioned, neither the initial population size.
12. Were those subjects who were prepared to participate representative of the entire population from which they were recruited?	Unable to determine			No mention of the proportion of those who agreed to participate. If so, no mention of the proportion of the participants that were excluded after data collection.
**INTERNAL VALIDITY – BIAS**				
15. Was an attempt made to blind those measuring the main outcomes of the intervention?	No			No blinding.
16. If any of the results of the study were based on "data dredging", was this made clear?		Yes		
18. Were the statistical tests used to assess the main outcomes appropriate?		Yes		
20. Were the main outcome measures used accurate (valid and reliable)?		Yes		
**INTERNAL VALIDITY – CONFOUNDING (SELECTION BIAS)**				

21. Were the patients in different intervention groups (trials and cohort studies) or were the cases and controls (case-control studies) recruited from the same population?	Unable to determin e			The source of patients is not provided.
22. Were study subjects in different intervention groups (trials and cohort studies) or were the cases and controls (case-control studies) recruited over the same period of time?	Unable to determin e			Unable to determine the timeline of the recruitment.
25. Was there adequate adjustment for confounding in the analyses from which the main findings were drawn?		Yes		

**Reference**	**Hamilton et al., 2020**			**Notes, justification**
**REPORTING**	**0**	**1**	**2**	
1. Is the hypothesis/aim/objective of the study clearly described?		Yes		
2. Are the main outcomes to be measured clearly described in the introduction or methods?	No			Authors don't describe the self-reported instability measurement (no question + no description of the Likert scale). The tool used for the quantitative laxity measurement is not described (while they give threshold values).
3. Are the characteristics of the patients included in the study clearly described?		Yes		
5. Are the distribution of principal confounders in each group of subjects to be compared clearly described?	No			No list of confounders is given.
6. Are the main findings of the study clearly described?		Yes		
7. Does the study provide estimates of the random variability in the data for the main outcomes?		Yes		
10. Have actual probability values been reported (*e.g. 0.035 rather than <0.05*) for the main outcomes except where the probability value is less than 0.001?		Yes		
**EXTERNAL VALIDITY**				
11. Were the subjects asked to participate in the study representative of the entire population from which they were recruited?	No			Source of population was not mentioned, neither the initial population size. Inclusion and exclusion criteria are not clearly described.
12. Were those subjects who were prepared to participate representative of the entire population from which they were recruited?		Yes		
**INTERNAL VALIDITY – BIAS**				
15. Was an attempt made to blind those measuring the main outcomes of the intervention?	No			No blinding.
16. If any of the results of the study were based on "data dredging", was this made clear?		Yes		
18. Were the statistical tests used to assess the main outcomes appropriate?		Yes		
20. Were the main outcome measures used accurate (valid and reliable)?		Yes		
**INTERNAL VALIDITY – CONFOUNDING (SELECTION BIAS)**				
21. Were the patients in different intervention groups (trials and cohort studies) or were the cases and controls (case-control studies) recruited from the same population?		Yes		
22. Were study subjects in different intervention groups (trials and cohort studies) or were the cases and controls (case-control studies) recruited over the same period of time?		Yes		
25. Was there adequate adjustment for confounding in the analyses from which the main findings were drawn?	No			No adjustment for the confounders in the analyses.

Reference	Knoop et al., 2012			Notes, justification
**REPORTING**	**0**	**1**	**2**	
1. Is the hypothesis/aim/objective of the study clearly described?		Yes		
2. Are the main outcomes to be measured clearly described in the introduction or methods?		Yes		
3. Are the characteristics of the patients included in the study clearly described?		Yes		
5. Are the distribution of principal confounders in each group of subjects to be compared clearly described?			Yes	The confounders are sex, age, BMI, radiographic severity, malalignment, stabilizing walking device, knee pain, pain medication, duration of knee. Symptoms.
6. Are the main findings of the study clearly described?		Yes		
7. Does the study provide estimates of the random variability in the data for the main outcomes?		Yes		
10. Have actual probability values been reported (*e.g. 0.035 rather than <0.05*) for the main outcomes except where the probability value is less than 0.001?		Yes		
**EXTERNAL VALIDITY**				
11. Were the subjects asked to participate in the study representative of the entire population from which they were recruited?	Unable to determine			No mention of the initial total size of population before applying the inclusion and exclusion criteria
12. Were those subjects who were prepared to participate representative of the entire population from which they were recruited?	Unable to determine			No mention of the proportion of those who agreed to participate. If so, no mention of the proportion of the participants that were excluded after data collection.
**INTERNAL VALIDITY – BIAS**				
15. Was an attempt made to blind those measuring the main outcomes of the intervention?	No			No blinding.
16. If any of the results of the study were based on "data dredging", was this made clear?		Yes		
18. Were the statistical tests used to assess the main outcomes appropriate?		Yes		
20. Were the main outcome measures used accurate (valid and reliable)?		Yes		
**INTERNAL VALIDITY – CONFOUNDING (SELECTION BIAS)**				
21. Were the patients in different intervention groups (trials and cohort studies) or were the cases and controls (case-control studies) recruited from the same population?		Yes		
22. Were study subjects in different intervention groups (trials and cohort studies) or were the cases and controls (case-control studies) recruited over the same period of time?	Unable to determine			Unable to determine the timeline of the recruitment.
25. Was there adequate adjustment for confounding in the analyses from which the main findings were drawn?		Yes		

Reference	Lewek et al., 2005			Notes, justification
**REPORTING**	**0**	**1**	**2**	
1. Is the hypothesis/aim/objective of the study clearly described?		Yes		
2. Are the main outcomes to be measured clearly described in the introduction or methods?		Yes		
3. Are the characteristics of the patients included in the study clearly described?		Yes		
5. Are the distribution of principal confounders in each group of subjects to be compared clearly described?	No			No list of confounders is given.
6. Are the main findings of the study clearly described?		Yes		

7. Does the study provide estimates of the random variability in the data for the main outcomes?		Yes		
10. Have actual probability values been reported (*e.g. 0.035 rather than <0.05*) for the main outcomes except where the probability value is less than 0.001?		Yes		
**EXTERNAL VALIDITY**				
11. Were the subjects asked to participate in the study representative of the entire population from which they were recruited?	No			Source of population was not clearly mentioned, neither the initial population size.
12. Were those subjects who were prepared to participate representative of the entire population from which they were recruited?	Unable to determine			No mention of the proportion of those who agreed to participate. If so, no mention of the proportion of the participants that were excluded after data collection.
**INTERNAL VALIDITY – BIAS**				
15. Was an attempt made to blind those measuring the main outcomes of the intervention?	No			No blinding.
16. If any of the results of the study were based on "data dredging", was this made clear?		Yes		
18. Were the statistical tests used to assess the main outcomes appropriate?		Yes		
20. Were the main outcome measures used accurate (valid and reliable)?		Yes		
**INTERNAL VALIDITY – CONFOUNDING (SELECTION BIAS)**				
21. Were the patients in different intervention groups (trials and cohort studies) or were the cases and controls (case-control studies) recruited from the same population?	Unable to determine			The source of patients is not provided.
22. Were study subjects in different intervention groups (trials and cohort studies) or were the cases and controls (case-control studies) recruited over the same period of time?	Unable to determine			Unable to determine the timeline of the recruitment.
25. Was there adequate adjustment for confounding in the analyses from which the main findings were drawn?	No			No adjustment for the confounders in the analyses.

**Reference**	**Rao et al., 2023**			**Notes, justification**
**REPORTING**	**0**	**1**	**2**	
1. Is the hypothesis/aim/objective of the study clearly described?		Yes		
2. Are the main outcomes to be measured clearly described in the introduction or methods?		Yes		
3. Are the characteristics of the patients included in the study clearly described?		Yes		
5. Are the distribution of principal confounders in each group of subjects to be compared clearly described?	No			No list of confounders is given.
6. Are the main findings of the study clearly described?		Yes		
7. Does the study provide estimates of the random variability in the data for the main outcomes?		Yes		
10. Have actual probability values been reported (*e.g. 0.035 rather than <0.05*) for the main outcomes except where the probability value is less than 0.001?		Yes		
**EXTERNAL VALIDITY**				
11. Were the subjects asked to participate in the study representative of the entire population from which they were recruited?	No			Source of population was not clearly mentioned, neither the initial population size.
12. Were those subjects who were prepared to participate representative of the entire population from which they were recruited?	Unable to determine			No mention of the proportion of those who agreed to participate. If so, no mention of the proportion of the participants that were excluded after data collection.
**INTERNAL VALIDITY – BIAS**				

15. Was an attempt made to blind those measuring the main outcomes of the intervention?	No			No blinding.
16. If any of the results of the study were based on "data dredging", was this made clear?		Yes		
18. Were the statistical tests used to assess the main outcomes appropriate?		Yes		
20. Were the main outcome measures used accurate (valid and reliable)?		Yes		
**INTERNAL VALIDITY – CONFOUNDING (SELECTION BIAS)**				
21. Were the patients in different intervention groups (trials and cohort studies) or were the cases and controls (case-control studies) recruited from the same population?	Unable to determine			The source of patients is not provided.
22. Were study subjects in different intervention groups (trials and cohort studies) or were the cases and controls (case-control studies) recruited over the same period of time?	Unable to determine			Unable to determine the timeline of the recruitment.
25. Was there adequate adjustment for confounding in the analyses from which the main findings were drawn?	No			No adjustment for the confounders in the analyses.

**Reference**	**Sanchez-Ramirez et al., 2016**			**Notes, justification**
**REPORTING**	**0**	**1**	**2**	
1. Is the hypothesis/aim/objective of the study clearly described?		Yes		
2. Are the main outcomes to be measured clearly described in the introduction or methods?		Yes		
3. Are the characteristics of the patients included in the study clearly described?		Yes		
5. Are the distribution of principal confounders in each group of subjects to be compared clearly described?	No			No list of confounders is given.
6. Are the main findings of the study clearly described?		Yes		
7. Does the study provide estimates of the random variability in the data for the main outcomes?		Yes		
10. Have actual probability values been reported (*e.g. 0.035 rather than <0.05*) for the main outcomes except where the probability value is less than 0.001?		Yes		
**EXTERNAL VALIDITY**				
11. Were the subjects asked to participate in the study representative of the entire population from which they were recruited?	Unable to determine			No mention of the initial total size of population before applying the inclusion and exclusion criteria
12. Were those subjects who were prepared to participate representative of the entire population from which they were recruited?	Unable to determine			No mention of the proportion of those who agreed to participate. If so, no mention of the proportion of the participants that were excluded after data collection.
**INTERNAL VALIDITY – BIAS**				
15. Was an attempt made to blind those measuring the main outcomes of the intervention?	No			No blinding.
16. If any of the results of the study were based on "data dredging", was this made clear?		Yes		
18. Were the statistical tests used to assess the main outcomes appropriate?		Yes		
20. Were the main outcome measures used accurate (valid and reliable)?		Yes		
**INTERNAL VALIDITY – CONFOUNDING (SELECTION BIAS)**				
21. Were the patients in different intervention groups (trials and cohort studies) or were the cases and controls (case-control studies) recruited from the same population?		Yes		
22. Were study subjects in different intervention groups (trials and cohort studies) or were the cases and controls (case-control studies) recruited over the same period of time?	Unable to determine			Unable to determine the timeline of the recruitment.

25. Was there adequate adjustment for confounding in the analyses from which the main findings were drawn?	No			No adjustment for the confounders in the analyses.

**Reference**	**Schmitt and Rudolph, 2008**			**Notes, justification**
**REPORTING**	**0**	**1**	**2**	
1. Is the hypothesis/aim/objective of the study clearly described?		Yes		
2. Are the main outcomes to be measured clearly described in the introduction or methods?		Yes		
3. Are the characteristics of the patients included in the study clearly described?		Yes		
5. Are the distribution of principal confounders in each group of subjects to be compared clearly described?	No			No list of confounders is given.
6. Are the main findings of the study clearly described?				
7. Does the study provide estimates of the random variability in the data for the main outcomes?		Yes		
10. Have actual probability values been reported (*e.g. 0.035 rather than* <*0.05*) for the main outcomes except where the probability value is less than 0.001?		Yes		
**EXTERNAL VALIDITY**				
11. Were the subjects asked to participate in the study representative of the entire population from which they were recruited?	Unable to determine			Source of population was not clearly mentioned, neither the initial population size before applying inclusion and exclusion criteria.
12. Were those subjects who were prepared to participate representative of the entire population from which they were recruited?	Unable to determine			No mention of the proportion of those who agreed to participate. If so, no mention of the proportion of the participants that were excluded after data collection.
**INTERNAL VALIDITY – BIAS**				
15. Was an attempt made to blind those measuring the main outcomes of the intervention?	No			No blinding.
16. If any of the results of the study were based on “data dredging”, was this made clear?		Yes		
18. Were the statistical tests used to assess the main outcomes appropriate?		Yes		
20. Were the main outcome measures used accurate (valid and reliable)?		Yes		
**INTERNAL VALIDITY – CONFOUNDING (SELECTION BIAS)**				
21. Were the patients in different intervention groups (trials and cohort studies) or were the cases and controls (case-control studies) recruited from the same population?		Yes		
22. Were study subjects in different intervention groups (trials and cohort studies) or were the cases and controls (case-control studies) recruited over the same period of time?	Unable to determine			Unable to determine the timeline of the recruitment.
25. Was there adequate adjustment for confounding in the analyses from which the main findings were drawn?		Yes		

**Reference**	**Schmitt et al., 2008**			**Notes, justification**
**REPORTING**	**0**	**1**	**2**	
1. Is the hypothesis/aim/objective of the study clearly described?		Yes		
2. Are the main outcomes to be measured clearly described in the introduction or methods?		Yes		
3. Are the characteristics of the patients included in the study clearly described?		Yes		
5. Are the distribution of principal confounders in each group of subjects to be compared clearly described?	No			No list of confounders is given.
6. Are the main findings of the study clearly described?		Yes		

7. Does the study provide estimates of the random variability in the data for the main outcomes?		Yes		
10. Have actual probability values been reported (*e.g. 0.035 rather than <0.05*) for the main outcomes except where the probability value is less than 0.001?		Yes		
**EXTERNAL VALIDITY**				
11. Were the subjects asked to participate in the study representative of the entire population from which they were recruited?	Unable to determine			Source of population was not clearly mentioned, neither the initial population size before applying inclusion and exclusion criteria.
12. Were those subjects who were prepared to participate representative of the entire population from which they were recruited?	Unable to determine			No mention of the proportion of those who agreed to participate. If so, no mention of the proportion of the participants that were excluded after data collection.
**INTERNAL VALIDITY – BIAS**				
15. Was an attempt made to blind those measuring the main outcomes of the intervention?	No			No blinding.
16. If any of the results of the study were based on "data dredging", was this made clear?		Yes		
18. Were the statistical tests used to assess the main outcomes appropriate?		Yes		
20. Were the main outcome measures used accurate (valid and reliable)?		Yes		
**INTERNAL VALIDITY – CONFOUNDING (SELECTION BIAS)**				
21. Were the patients in different intervention groups (trials and cohort studies) or were the cases and controls (case-control studies) recruited from the same population?	Unable to determine			The source of patients is not provided.
22. Were study subjects in different intervention groups (trials and cohort studies) or were the cases and controls (case-control studies) recruited over the same period of time?	Unable to determine			Unable to determine the timeline of the recruitment.
25. Was there adequate adjustment for confounding in the analyses from which the main findings were drawn?	No			No adjustment for the confounders in the analyses.

**Reference**	**Schrijvers et al., 2021**			**Notes, justification**
**REPORTING**	**0**	**1**	**2**	
1. Is the hypothesis/aim/objective of the study clearly described?		Yes		
2. Are the main outcomes to be measured clearly described in the introduction or methods?		Yes		
3. Are the characteristics of the patients included in the study clearly described?		Yes		
5. Are the distribution of principal confounders in each group of subjects to be compared clearly described?	No			No list of confounders is given.
6. Are the main findings of the study clearly described?		Yes		
7. Does the study provide estimates of the random variability in the data for the main outcomes?		Yes		Data obtained from the supplementary materials.
10. Have actual probability values been reported (*e.g. 0.035 rather than <0.05*) for the main outcomes except where the probability value is less than 0.001?		Yes		
**EXTERNAL VALIDITY**				
11. Were the subjects asked to participate in the study representative of the entire population from which they were recruited?	Unable to determine			No mention of the initial total size of population before applying the inclusion and exclusion criteria.
12. Were those subjects who were prepared to participate representative of the entire population from which they were recruited?	Unable to determine			No mention of the proportion of those who agreed to participate. If so, no mention of the proportion of the participants that were excluded after data collection.
**INTERNAL VALIDITY – BIAS**				

15. Was an attempt made to blind those measuring the main outcomes of the intervention?	No			No blinding.
16. If any of the results of the study were based on "data dredging", was this made clear?		Yes		
18. Were the statistical tests used to assess the main outcomes appropriate?		Yes		
20. Were the main outcome measures used accurate (valid and reliable)?		Yes		
**INTERNAL VALIDITY – CONFOUNDING (SELECTION BIAS)**				
21. Were the patients in different intervention groups (trials and cohort studies) or were the cases and controls (case-control studies) recruited from the same population?		Yes		
22. Were study subjects in different intervention groups (trials and cohort studies) or were the cases and controls (case-control studies) recruited over the same period of time?	Unable to determine			Unable to determine the timeline of the recruitment.
25. Was there adequate adjustment for confounding in the analyses from which the main findings were drawn?	No			No adjustment for the confounders in the analyses.

**Reference**	**Segal et al., 2015**			**Notes, justification**
**REPORTING**	**0**	**1**	**2**	
1. Is the hypothesis/aim/objective of the study clearly described?		Yes		
2. Are the main outcomes to be measured clearly described in the introduction or methods?		Yes		
3. Are the characteristics of the patients included in the study clearly described?		Yes		
5. Are the distribution of principal confounders in each group of subjects to be compared clearly described?		Partially		Confounders are not mentioned explicitly. Age, knee pain, varus alignment, knee surgery, knee injury history.
6. Are the main findings of the study clearly described?		Yes		
7. Does the study provide estimates of the random variability in the data for the main outcomes?		Yes		
10. Have actual probability values been reported (*e.g. 0.035 rather than <0.05*) for the main outcomes except where the probability value is less than 0.001?		Yes		
**EXTERNAL VALIDITY**				
11. Were the subjects asked to participate in the study representative of the entire population from which they were recruited?		Yes		
12. Were those subjects who were prepared to participate representative of the entire population from which they were recruited?		Yes		
**INTERNAL VALIDITY – BIAS**				
15. Was an attempt made to blind those measuring the main outcomes of the intervention?	No			No blinding.
16. If any of the results of the study were based on "data dredging", was this made clear?		Yes		
18. Were the statistical tests used to assess the main outcomes appropriate?		Yes		
20. Were the main outcome measures used accurate (valid and reliable)?		Yes		
**INTERNAL VALIDITY – CONFOUNDING (SELECTION BIAS)**				
21. Were the patients in different intervention groups (trials and cohort studies) or were the cases and controls (case-control studies) recruited from the same population?		Yes		
22. Were study subjects in different intervention groups (trials and cohort studies) or were the cases and controls (case-control studies) recruited over the same period of time?		Yes		

25. Was there adequate adjustment for confounding in the analyses from which the main findings were drawn?		Yes		

Reference	Shakoor et al., 2017			Notes, justification
**REPORTING**	**0**	**1**	**2**	
1. Is the hypothesis/aim/objective of the study clearly described?		Yes		
2. Are the main outcomes to be measured clearly described in the introduction or methods?		Yes		
3. Are the characteristics of the patients included in the study clearly described?		Yes		
5. Are the distribution of principal confounders in each group of subjects to be compared clearly described?			Yes	The confounders are age, sex, BMI, race, KL grade, hip/ankle/foot pain, knee pain, vibratory perception threshold, strength.
6. Are the main findings of the study clearly described?		Yes		
7. Does the study provide estimates of the random variability in the data for the main outcomes?		Yes		
10. Have actual probability values been reported (*e.g. 0.035 rather than <0.05*) for the main outcomes except where the probability value is less than 0.001?		Yes		
**EXTERNAL VALIDITY**				
11. Were the subjects asked to participate in the study representative of the entire population from which they were recruited?		Yes		
12. Were those subjects who were prepared to participate representative of the entire population from which they were recruited?		Yes		
**INTERNAL VALIDITY – BIAS**				
15. Was an attempt made to blind those measuring the main outcomes of the intervention?	No			No blinding.
16. If any of the results of the study were based on "data dredging", was this made clear?		Yes		
18. Were the statistical tests used to assess the main outcomes appropriate?		Yes		
20. Were the main outcome measures used accurate (valid and reliable)?		Yes		
**INTERNAL VALIDITY – CONFOUNDING (SELECTION BIAS)**				
21. Were the patients in different intervention groups (trials and cohort studies) or were the cases and controls (case-control studies) recruited from the same population?		Yes		
22. Were study subjects in different intervention groups (trials and cohort studies) or were the cases and controls (case-control studies) recruited over the same period of time?		Yes		
25. Was there adequate adjustment for confounding in the analyses from which the main findings were drawn?		Yes		

Reference	Sharma et al., 2015			Notes, justification
**REPORTING**	**0**	**1**	**2**	
1. Is the hypothesis/aim/objective of the study clearly described?		Yes		
2. Are the main outcomes to be measured clearly described in the introduction or methods?		Yes		
3. Are the characteristics of the patients included in the study clearly described?		Yes		
5. Are the distribution of principal confounders in each group of subjects to be compared clearly described?			Yes	The confounders are age, sex, BMI, race, KL grade, knee pain severity, function self-efficacy, depressive symptoms, disease severity, knee extensor strength.
6. Are the main findings of the study clearly described?		Yes		
7. Does the study provide estimates of the random variability in the data for the main outcomes?		Yes		

10. Have actual probability values been reported (*e.g. 0.035 rather than <0.05*) for the main outcomes except where the probability value is less than 0.001?	No			Don't give any p-value.
**EXTERNAL VALIDITY**				
11. Were the subjects asked to participate in the study representative of the entire population from which they were recruited?	Unable to determine			No mention of the initial total size of population before applying the inclusion and exclusion criteria.
12. Were those subjects who were prepared to participate representative of the entire population from which they were recruited?		Yes		
**INTERNAL VALIDITY – BIAS**				
15. Was an attempt made to blind those measuring the main outcomes of the intervention?	No			No blinding.
16. If any of the results of the study were based on "data dredging", was this made clear?		Yes		
18. Were the statistical tests used to assess the main outcomes appropriate?		Yes		
20. Were the main outcome measures used accurate (valid and reliable)?		Yes		
**INTERNAL VALIDITY – CONFOUNDING (SELECTION BIAS)**				
21. Were the patients in different intervention groups (trials and cohort studies) or were the cases and controls (case-control studies) recruited from the same population?		Yes		
22. Were study subjects in different intervention groups (trials and cohort studies) or were the cases and controls (case-control studies) recruited over the same period of time?	Unable to determine			Unable to determine the timeline of the recruitment.
25. Was there adequate adjustment for confounding in the analyses from which the main findings were drawn?		Yes		

**Reference**	**Skou et al., 2014**			**Notes, justification**
**REPORTING**	**0**	**1**	**2**	
1. Is the hypothesis/aim/objective of the study clearly described?		Yes		
2. Are the main outcomes to be measured clearly described in the introduction or methods?		Yes		
3. Are the characteristics of the patients included in the study clearly described?		Yes		
5. Are the distribution of principal confounders in each group of subjects to be compared clearly described?		Partially		Confounders are not explicitly described. Age and sex.
6. Are the main findings of the study clearly described?		Yes		
7. Does the study provide estimates of the random variability in the data for the main outcomes?		Yes		
10. Have actual probability values been reported (*e.g. 0.035 rather than <0.05*) for the main outcomes except where the probability value is less than 0.001?		Yes		
**EXTERNAL VALIDITY**				
11. Were the subjects asked to participate in the study representative of the entire population from which they were recruited?	Unable to determine			No mention of the initial total size of population before applying the inclusion and exclusion criteria.
12. Were those subjects who were prepared to participate representative of the entire population from which they were recruited?	Unable to determine			No mention of the proportion of those who agreed to participate. If so, no mention of the proportion of the participants that were excluded after data collection.
**INTERNAL VALIDITY – BIAS**				
15. Was an attempt made to blind those measuring the main outcomes of the intervention?	No			No blinding.

16. If any of the results of the study were based on "data dredging", was this made clear?		Yes		
18. Were the statistical tests used to assess the main outcomes appropriate?		Yes		
20. Were the main outcome measures used accurate (valid and reliable)?		Yes		
**INTERNAL VALIDITY – CONFOUNDING (SELECTION BIAS)**				
21. Were the patients in different intervention groups (trials and cohort studies) or were the cases and controls (case-control studies) recruited from the same population?	Unable to determine			The source of patients is not provided.
22. Were study subjects in different intervention groups (trials and cohort studies) or were the cases and controls (case-control studies) recruited over the same period of time?	Unable to determine			Unable to determine the timeline of the recruitment.
25. Was there adequate adjustment for confounding in the analyses from which the main findings were drawn?	No			No adjustment for the confounders in the analyses.

**Reference**	**Van der Esch et al., 2006**			**Notes, justification**
**REPORTING**	**0**	**1**	**2**	
1. Is the hypothesis/aim/objective of the study clearly described?		Yes		
2. Are the main outcomes to be measured clearly described in the introduction or methods?		Yes		
3. Are the characteristics of the patients included in the study clearly described?		Yes		
5. Are the distribution of principal confounders in each group of subjects to be compared clearly described?	No			No list of confounders is given.
6. Are the main findings of the study clearly described?		Yes		
7. Does the study provide estimates of the random variability in the data for the main outcomes?		Yes		
10. Have actual probability values been reported (*e.g. 0.035 rather than <0.05*) for the main outcomes except where the probability value is less than 0.001?		Yes		
**EXTERNAL VALIDITY**				
11. Were the subjects asked to participate in the study representative of the entire population from which they were recruited?	No			Source of population was not clearly mentioned, neither the initial population size before applying inclusion and exclusion criteria.
12. Were those subjects who were prepared to participate representative of the entire population from which they were recruited?	Unable to determine			No mention of the proportion of those who agreed to participate. If so, no mention of the proportion of the participants that were excluded after data collection.
**INTERNAL VALIDITY – BIAS**				
15. Was an attempt made to blind those measuring the main outcomes of the intervention?	No			No blinding.
16. If any of the results of the study were based on "data dredging", was this made clear?		Yes		
18. Were the statistical tests used to assess the main outcomes appropriate?		Yes		
20. Were the main outcome measures used accurate (valid and reliable)?		Yes		
**INTERNAL VALIDITY – CONFOUNDING (SELECTION BIAS)**				
21. Were the patients in different intervention groups (trials and cohort studies) or were the cases and controls (case-control studies) recruited from the same population?	Unable to determine			The source of patients is not provided.
22. Were study subjects in different intervention groups (trials and cohort studies) or were the cases and	Unable to			Unable to determine the timeline of the recruitment.

controls (case-control studies) recruited over the same period of time?	determi ne			
25. Was there adequate adjustment for confounding in the analyses from which the main findings were drawn?	No			No adjustment for the confounders in the analyses.

Summary of studies included in the systematic review

In total, 738 articles were identified from the systematic research. Thirty-four articles were assessed for eligibility. Three additional articles were identified through reference lists. We included 19 articles in the systematic review. A summary of the outcomes of interest is presented in Table S3.

Table S3. Summary of studies included in the systematic review

Reference	Type	$N_{unstable}$	N_{stable}	Stability measurement	Neuromuscular function assessment	Results
Chaudhari et al. (2019)	OA	20	15	"To what degree does giving way, buckling, or shifting of your knee affect your level of daily activity?" Score from 0 (symptoms the prevent all activity) to 5 (no symptoms) (KOS-ADLS, Irrgang et al., 1998)<br><br>Varus-Valgus laxity during surgery (load of 10Nm)	MVC, Isom, 60° of flexion, Q. Normalized to body mass.	Significantly less Q strength between stable and unstable groups (p = 0.001). A lower muscle strength is associated with a greater knee instability (p = 0.01).
Farrokhi et al. (2015)	OA	17	36	KOS-ADLS	MVC, Isom, 60° of flexion, Q. Normalized to body mass.	Significantly worse self-reported knee stiffness in unstable compared to stable patients (p < 0.01). No significant difference in Q strength (p = 0.28).
Felson et al. (2007)	OA	278	2073	"Have you had an episode in the past 3 months where your knee buckled or gave way?". If the answer is yes, specify which knee, the frequency, if it precipitated a fall,	MVC Isom, Q. Normalized to body weight.	Q strength is associated with buckling risk (independent of age, sex, BMI) (OR 0.027, 95% CI 0.006-0.122).

				the activity they were doing when it occurred.		
Fleeton et al. (2016)	TKR	74	239	KOS-ADL	MVC, Isom, 90° of flexion, Q, and H. Handheld dynamometer. Normalized to body mass. Stair-climb power test.	6-weeks post-op: unstable TKR have significantly less Q strength compared to stables ($p \leq 0.05$). No significant differences for H strength ($p = 0.24$). Unstable TKR have a reduced lower-limb power compared to stables ($p < 0.05$). 6-months post-op: unstable TKR have no significant difference in Q and H strength compared to stables ($p = 0.17$; $p = 0.44$). Unstable TKR have a reduced lower-limb power compared to stables ($p < 0.05$). Significant univariate associations between 6-weeks stair power (<150 watts) (OR 3.24, 95% CI 1.81-5.82, $p < 0.01$) and retained knee instability 6-months post-op. No association with 6-weeks H strength (<0.34 N/m/kg) and Q strength (<0.54 Nm/kg) (OR 1.64, 95% CI 0.93-2.91, $p \leq 0.1$) and retained knee instability 6-months post-op (OR 1.12, 95% CI 0.63-1.98, $p = 0.7$).
Freisinger et al. (2017a)	OA	Unclear	Unclear	KOS-ADLS<br><br>Varus-Valgus laxity during surgery (load of 10Nm)	MVC, Isom, 60° of flexion, Q, and H. Normalized to body mass.	Varus-valgus laxity significantly correlates with Q strength ($r_P = 0.444$, $p = 0.014$) but not with hamstrings strength ($r_P = 0.225$, $p = 0.233$). In the multivariate general regression model, only Q strength showed a trend with varus-valgus laxity ($R^2 = 0.29$, adj $R^2 = 0.17$, $p = 0.053$).

Gustafson et al. (2016)	OA	17	35	KOS-ADLS<br><br>Medial compartment joint laxity (TELOS device, 150Nm, knee flexed at 20°)	Walking knee stiffness during weight acceptance. MVC, Isom, Q, 60° of flexion. Normalized to body weight. Self-reported knee stiffness using the WOMAC.	No significant difference in Q strength between stable and unstable groups (p = 0.34). Unstable subjects have a significantly worse self-reported knee stiffness (p < 0.01), but a lower walking joint stiffness compared to the stables (p = 0.01). No significant association between knee stiffness and medial laxity (R^2 = 0.03, p = 0.094).
Hamilton et al. (2020)	TKR	0 (self-reported) Loose: 9 coronal/ 10 sagittal	42 Neutral: 22 coronal/17 sagittal Tight: 11/15	Clinical physical exam in coronal and sagittal planes (medial/lateral stress at 30° of flexion, anterior drawer test at 90° of flexion). Tight: no joint opening, neutral: 5mm of joint opening, loose: 10mm of joint opening. Feelings of instability (5-points Likert scale).	Lower limb power measured with a leg extensor power rig.	No significant difference between laxity and power (p = 0.23 for coronal plane, p = 0.98 for sagittal plane).
Knoop et al. (2012)	OA	191	92	"Have you had an episode in the past 3 months where your knee buckled or gave way?". If the answer is yes, specify which knee, the frequency, if it precipitated a fall, the activity they were doing when it occurred.<br><br>Laxity (weight on the lower leg)	MVC, Isok, 60°/s, Q, and H. Normalized to body mass.	Significantly less muscle strength between stable and unstable groups (p ≤ 0.001). Higher muscle strength is significantly associated with the absence of knee instability (crude OR 0.28, 95% CI 0.15-0.52, p < 0.001). No effect modification could be demonstrated between muscle strength and laxity (OR 1.01, 95% IC 0.87-1.18, p = 0.88).
Lewek et al. (2005)	OA	17	4	KOS-ADLS<br><br>Frontal plane laxity (TELOS device,	Co-contraction with the OA leg on a movable platform, *VM*-MH, *VL*-LH,	Prior to translation, stable patients have a significantly larger VM-MH co-contraction than the unstable group (p = 0.038). In OA subjects, higher

				150Nm, knee flexed at 20°)	*VM*-MG, *VL*-LG. Normalized to %MVC isometric.	VM-MH co-contraction is significantly correlated with greater stability when there are no translations (r_s = 0.459, p = 0.042).
Rao et al. (2023)	TKR	8	10	Patients reporting episodes of buckling, shifting, or giving away during daily activities in the 3-months prior to recruitment, with or without clinical signs (manual passive laxity tests + PROMs) of instability.<br><br>Report any feelings of instability on a 4-point scale after each activity.	Muscular activations (RF, VM, VL, MH, LH, TA, MG, LG) during level and downhill walking, and stair descent. Muscle synergies.	Unstable patients demonstrated a heterogeneous muscle synergy response, with prolonged activation of dorsi- and knee flexor muscles (effect size = 1.49, p = 0.01) during stair descent. Knee extensor-flexor co-activation on both groups through stance phase. High contribution of the knee flexor muscles during late swing/early stance for all activities.
Sanchez-Ramirez et al. (2016)	OA	7	26	"Have you had an episode in the past 3 months where your knee buckled or gave way?". If the answer is yes, specify which knee, the frequency, if it precipitated a fall, the activity they were doing when it occurred.	MVC, Isok 60°/s, Isom 60°, Q, and H. Normalized to body weight. Co-contraction during a step-down task, *VM*-MH, *VL*-LH, and *VL*-MH.	Unstable patients have a significantly different Isom (p = 0.049) and Isok (p = 0.031) lower muscle strength compared to stables. No significant difference in muscle activity and co-contraction between stable and unstable groups during the loading phase of a step-down task. No significant correlation between muscle activity and co-contraction and stability.
Schmitt et al. (2008)	OA	32	20	KOS-ADLS<br><br>Frontal plane laxity (TELOS device, 150Nm, knee flexed at 20°)	MVC, Isom, 60° of flexion, Q. Normalized to height.	No significant difference for Q strength between stable and unstable OA groups (p = 0.453). Knee instability is not related to Q muscle strength (Eta^2 = 0.032, p = 0.453).
Schmitt and	OA	10	10	KOS-ADLS	MVC, Isom, 90° of flexion, Q.	No significant difference for Q strength between stable and

Rudolph (2008)				Frontal plane laxity (TELOS device, 150Nm, knee flexed at 20°)	Co-contraction while walking across a movable platform, LQ-LH, LQ-LG, MQ-MH, MQ-MG. Normalized by peak muscle activity during undisturbed walking.	unstable OA groups ($p = 0.309$). Unstable OA use significantly higher MQ-MH during preparation ($p = 0.012$) and weight acceptance ($p = 0.005$); and MQ-MG during weight acceptance ($p = 0.033$). During single limb support, unstable patients used higher MQ-MH but it was not significant ($p = 0.086$). Instability score contributes significantly to predict MQ-MH co-contraction during the preparation phase ($R^2 = 0.269$, $p = 0.022$). Instability score contributes significantly to predict MQ-MH ($R^2 = 0.420$, $p = 0.006$) and MQ-MG ($R^2 = 0.360$, $p = 0.018$) co-contraction during weight acceptance. Instability experienced during testing does not correlate with MQ-MH during preparation ($r_s = -0.292$, $p = 0.256$) and weight acceptance ($r_s = -0.202$, $p = 0.436$); but correlates with MQ-MG during weight acceptance ($r_s = -0.5$, $p = 0.048$).
Schrijvers et al. (2021)	OA	20	20	"In the past 4 months, have you had an episode of buckling, shifting, or giving way?" If the answer is yes, the patient is considered unstable.	Treadmill, perturbed walking trials. Muscle activity (*VL, VM, RF,* LH, MH, LG, MG, *GM*). Co-contraction index: *VM-MH, VL*-LH and total of the knee. Normalized by peak muscle activity during	Overall, muscle activation is similar between stable and unstable. Some significant ($p \leq 0.05$) differences between groups and subgroups (*e.g.,* moderate OA-unstable vs. moderate OA-stable, moderate OA-unstable vs. severe OA-stable, moderate OA-unstable vs. severe OA-unstable, moderate, severe OA-unstable vs. moderate OA-unstable, severe OA-unstable vs. severe OA-stable).

					undisturbed walking.	
Segal et al. (2015)	OA	Unclear	Unclear	"In the past 3 months, has either of your knees buckled or gave way at least once?", "in the past 3 months, has either knee felt like it was shifting, slipping or going to give way but it didn't actually do so?". If the answer is yes, specify which knee and the frequency.	MH and LH co-activation during Q contraction. Measured during an Isok test, extension movement.	Results are only presented for overall instability. No significant association between both instability prevalence (baseline) and incidence (T24-months) and, combined, medial, and lateral hamstring co-activation after adjustments for covariates (sex, age, KL grade, knee pain).
Shakoor et al. (2017)	OA	652	1151	"In the past 3 months, has either of your knees buckled or gave way at least once?", "in the past 3 months, has either knee felt like it was shifting, slipping or going to give way but it didn't actually do so?". If the answer is yes, specify which knee and the frequency.	MVC, Isok, 60°/s, Q. Normalized to body size.	Results are only presented for overall instability. Greater Q strength is strongly associated with a decrease incidence (RR 0.53, 95% CI. 0.38-0.75, $p < 0.001$) and lower risk of worsening (RR 0.73, 95% CI 0.58-0.92, $p = 0.008$) of the instability symptoms. Significant linear trends between Q strength and instability incidence ($p \leq 0.001$) and worsening ($p = 0.01$).
Sharma et al. (2015)	OA	76	136	"Has your knee buckled or given way at least once in the past 3 months? Which knee buckled or gave way at least once (right, left, both, don't know)?"	MVC, Isok, 120°/s, Q. Normalized to body weight.	No analysis of the Q strength results (used as a covariate in the study). Patients reporting buckling have a Q muscle strength of 87.4 ± 32.7 Nm/kg, while patients not reporting buckling have a Q muscle strength of 95.9 ± 29.1 Nm/kg.
Skou et al. (2014)	OA	76	24	"Does your knee give way, buckle, or shift with ..." (strenuous activities, moderate activities, activities of daily living, unable to	MVC, Isom, 60° of flexion, Q. Normalized to body mass.	No significant association between Q strength and knee instability ($r_s = -0.21$, p = NS).

				perform any of those). 5 points Likert-scale.		
Van der Esch et al. (2006)	OA	Undefin ed	Undefined	Laxity (weight of 1.12kg the lower leg, knee flexed at 20°)	MVC, Isok, 60°/s, Q and H. Mean of right and left sides. Normalized to body weight.	Significant correlations between joint laxity and total muscle strength (r_P = -0.34, $p < 0.05$).

BMI: body mass index, CI: confidence interval, GM: gluteus medius, H: hamstrings, Isok: isokinetic, Isom: isometric, KOS-ADLS: Knee Outcome Survey-Activities of Daily Living Scale, LG: lateral gastrocnemius, LH: lateral hamstring, LQ: lateral quadriceps, MG: medial gastrocnemius, MH: medial hamstring, MQ: medial quadriceps, MVC: maximal voluntary contraction, NS: non-significant, OA: osteoarthritis, PROMs: patient-reported outcome measures, Q: quadriceps, r_s*: Spearman's rho, RF: rectus femoris, RR: relative risk, VL: vastus lateralis, VM: vastus lateralis.*

Summary of studies included in the meta-analyses

Ultimately, 11 studies could be included in the meta-analysis. Nine focused on muscle strength (Table S4), and 4 on muscle co-contraction (Table S5).

When authors used different measurements (*e.g.*, quadriceps and hamstrings, isometric/isokinetic), we only kept the most commonly used one. When authors measured co-contraction at different moments, we chose to only keep the data collected when no perturbation was occurring.

Table S4. Summary of included studies in the lower-limb muscle strength meta-analysis.

Reference	Type	Number of		Strength			
		Unstable	Stable	Measure	Muscle	Stable	Unstable
Chaudhari et al., 2019	OA	20	15	ISOM 60°	Q	1.26 ± 0.33 Nm/kg	0.79 ± 0.42 Nm/kg
Fleeton et al., 2016	TKR	74	239	ISOM 60°	Q	0.74±0.33* Nm/kg	0.80 ± 0.31 Nm/kg
Gustafson et al., 2016	OA	17	35	ISOM 60°	Q	1.9 ± 0.6 Nm/kg	1.7 ± 0.4 Nm/kg
Knoop et al., 2012	OA	191	92	ISOK 60°/s	General strength (Q H)	1.02 ± 0.39 Nm/kg	0.80 ± 0.41 Nm/kg
Sanchez-Ramirez et al., 2016	OA	7	26	ISOM 60°	General strength (Q H)	0.99 ± 0.05[a] Nm/kg	0.86 ± 0.09[a] Nm/kg
Schmitt and Rudolph, 2008	OA	10	10	ISOM 90°	Q	757 ± 231 N	651 ± 208 N
Schmitt et al., 2008	OA	32	20	ISOM 90°	Q	471.52 ± 130.92 N/m	433.81 ± 170.96* N/m
Sharma et al., 2015	OA	76	136	ISOK 120°/s	Q	95.9 ± 29.1 Nm/kg	87.4 ± 32.7 Nm/kg
Farrokhi et al., 2015	OA	17	36	ISOM 60°	Q	1.9 ± 0.6 Nm/kg	1.7 ± 0.4 Nm/kg

**: pooled means and standard deviation, [a]: values extracted from graph, H: hamstrings, ISOM: isometric, ISOK: isokinetic, OA: osteoarthritis, Q: quadriceps, TKR: total knee replacement.*
Only values used in the meta-analyses are reported. When subgroups existed, we pooled the means and standard deviations.

Table S5. Summary of included studies in the lower limbs muscle co-contraction meta-analysis.

Reference	Type	Number of					Muscle activity	
		Unstable	Stable	Measure	Muscle	Activity	Stable	Unstable
Lewek et al., 2005	OA	17	4	Co-contraction index	VMMH	Prior to perturbation (standing)	3.80 ± 3.08	1.67 ± 1.18

Sanchez-Ramirez et al., 2016	OA	7	26	Co-contraction index	VMMH	Step down task	0.57 ± 0.2	0.60 ± 0.2
Schmitt and Rudolph, 2008	OA	10	10	Co-contraction "value"	MQH	Prior to perturbation (walk)	48.7 ± 22.4[a]	73.6 ± 18.3[a]
Schrijvers et al., 2021	OA	20	20	Co-contraction "indices"	Medial muscles	After perturbation stride	0.7 ± 0.23	0.74 ± 0.14

[a]: values extracted from graph, MQH: medial quadriceps and hamstring, OA: osteoarthritis, VMMH: vastus medialis and medial hamstring.
Only values used in the different meta-analyses are reported.

References

1. Munn J, Sullivan SJ, Schneiders AG. Evidence of sensorimotor deficits in functional ankle instability: a systematic review with meta-analysis. *J Sci Med Sport*. 2010;13(1):2-12. doi:10.1016/j.jsams.2009.03.004
2. Downs SH, Black N. The feasibility of creating a checklist for the assessment of the methodological quality both of randomized and non-randomized studies of health care interventions. *J Epidemiol Community Health*. 1998;52(6):377-384. doi:10.1136/jech.52.6.377
